# Advances in Organic and Organic-Inorganic Hybrid Polymeric Supports for Catalytic Applications

**DOI:** 10.3390/molecules21101288

**Published:** 2016-09-28

**Authors:** Anna Maria Pia Salvo, Francesco Giacalone, Michelangelo Gruttadauria

**Affiliations:** Dipartimento Scienze e Tecnologie Biologiche, Chimiche e Farmaceutiche (STEBICEF) Università di Palermo, Viale delle Scienze, Ed. 17, 90128 Palermo, Italy; annamariapia.salvo@unipa.it (A.M.P.S.); francesco.giacalone@unipa.it (F.G.)

**Keywords:** heterogeneous catalysis, organocatalysis, metal catalyst, asymmetric catalysis, organic-inorganic hybrid, polystyrene

## Abstract

In this review, the most recent advances (2014–2016) on the synthesis of new polymer-supported catalysts are reported, focusing the attention on the synthetic strategies developed for their preparation. The polymer-supported catalysts examined will be organic-based polymers and organic-inorganic hybrids and will include, among others, polystyrenes, poly-ionic liquids, chiral ionic polymers, dendrimers, carbon nanotubes, as well as silica and halloysite-based catalysts. Selected examples will show the synthesis and application in the field of organocatalysis and metal-based catalysis both for non-asymmetric and asymmetric transformations.

## 1. Introduction

Polymer-supported catalysts are a topic of paramount importance. The continuously increasing number of publications on the topic demonstrates the interest of the scientific community toward the development of this kind of materials. Such kinds of catalytic materials present some advantages over their homogeneous counterparts, but also some disadvantages that, in some cases may limit their application in industry [1]. Their solubility profile may help both in the catalyst and in product recovery [2]. On the other hand, their higher cost, due to the longer synthetic strategies, the possible leaching of the active catalytic species with lower activity upon recycling, and their possible lower overall catalytic activity, may diminish their usefulness. Nevertheless, even considering a narrow timeframe (2014–2016) the number of publications is quite impressive and many efforts are currently devoted in order to avoid the above disadvantages. Therefore, we have focused our attention only on a selected number of papers and selected topics. A useful way to classify polymer-supported catalysts is based on the type of catalysed reactions or supports employed. Here, we have pointed the focus on the organocatalytic reactions and metal-catalysed reactions in order to highlight the synthetic importance in non-asymmetric and asymmetric transformations. Moreover, starting from the catalytic application of organic or organic-inorganic hybrid polymeric supported catalysts, we also discuss the new synthetic developments regarding the preparation of such polymeric supports.

## 2. Organocatalysed Non-Asymmetric Reactions

### 2.1. Brønsted Acid Catalysed Reactions

Acid-catalysed reactions represent one of the most important technologies applied in the chemical industry. Mineral acids, used in homogeneous phase reactions, usually show high catalytic activity, but they suffer several drawbacks, such as occurrence of side reactions, corrosion of the equipment and the production of large amounts of acidic wastes. These reactions require tedious procedures for isolation of the products, which could cause environmental issues. In order to improve catalyst separation and recycling, various solid catalysts are widely used. Among them, sulfonic acid group functionalized polymer materials exhibit outstanding activity and reasonable recyclability in several acid-catalysed organic reactions, including esterification and Beckmann reactions.

#### 2.1.1. Esterification Reactions

**Scheme 1:**
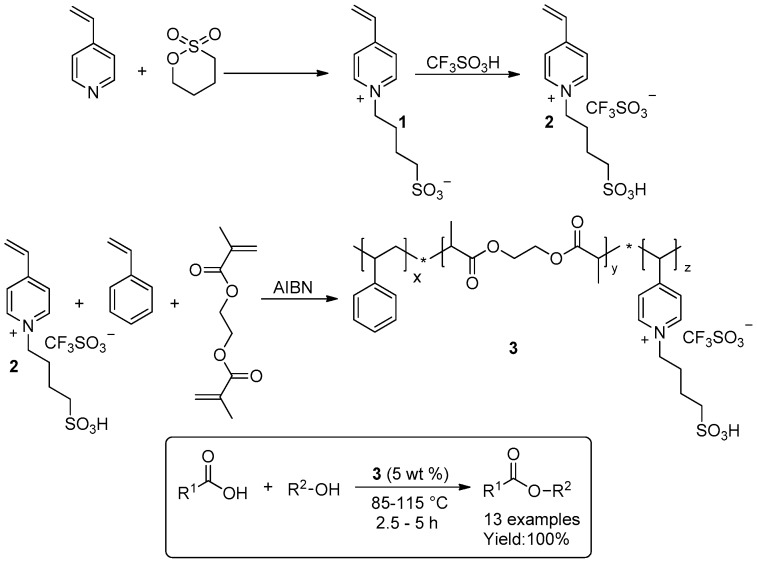
Synthesis of poly ionic liquid acid **3** and its catalytic activity in esterification reactions.

Recently, Zhang et al. reported the synthesis of poly-ionic liquid (PIL) acid **3** and its use as a catalyst in esterification reactions (Scheme 1) [3]. The acid **3** was obtained by polymerization of ionic liquid monomer **2** with styrene and ethyleneglycol dimethacrylate in the presence of azobisiso-butyronitrile (AIBN). Ionic liquid monomer **2** was prepared in two synthetic steps: (a) reaction between 4-vinylpyridine and 1,4-butylsultone; (b) treatment of intermediate **1** with trifluoromethane-sulfonic acid. Under optimized conditions, the poly-ionic liquid acid **3** was then used in the synthesis of various esters at appropriate reaction temperatures and times, and quantitative yields were achieved. The activity and stability of the catalyst were maintained after five recycles. Furthermore, the continuous formation of methyl methacrylate was investigated using a fixed bed reactor and the methyl methacrylate product was obtained in high yield (>96%).

**Scheme 2:**
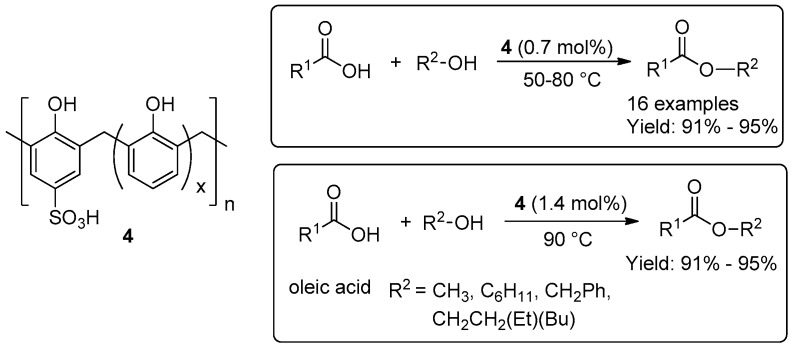
Esterification of carboxylic acids catalysed by resin **4**.

In 2013, Minakawa et al. reported the use of the macroporous phenolsulfonic acid-formaldehyde resin **4** as a heterogeneous polymeric acid catalyst in the direct dehydrative esterification of alcohols and carboxylic acids. The corresponding esters were obtained in 91%–95% yield without removal of water (Scheme 2) [4]. In 2016, the resin **4** was employed with excellent results (yields of 91%–95%) in the esterification of oleic acid with various alcohols and its recyclability was evaluated [5]. The intrinsic physical properties of resin **4** allowed increasing the yield by shifting the equilibrium toward esterification without the need for a water-removal procedure. In fact, the slightly hydrophilic meso-/macroporous structure of the resins could absorb fairly hydrophilic substrates and transform them into more hydrophobic esters, which in turn could readily be excluded from the catalyst surface. After esterification, the resin was filtered, washed with ethanol and treated with concentrated sulfuric acid in order to regenerate the active sulfonic acid sites. The catalyst was recyclable up to at least 30 times without any significant loss in its activity. Thanks to its surface properties and porous structure, resin **4** exhibited higher catalytic activity than a variety of commercially available heterogeneous solid-acid catalysts tested in the esterification of oleic acid and methanol (Montmorillonite K-10, Amberlyst 15, Amberlite IRA 400, sulfonated ZrO_2_, H-ZSM-5, PTSA).

**Scheme 3:**
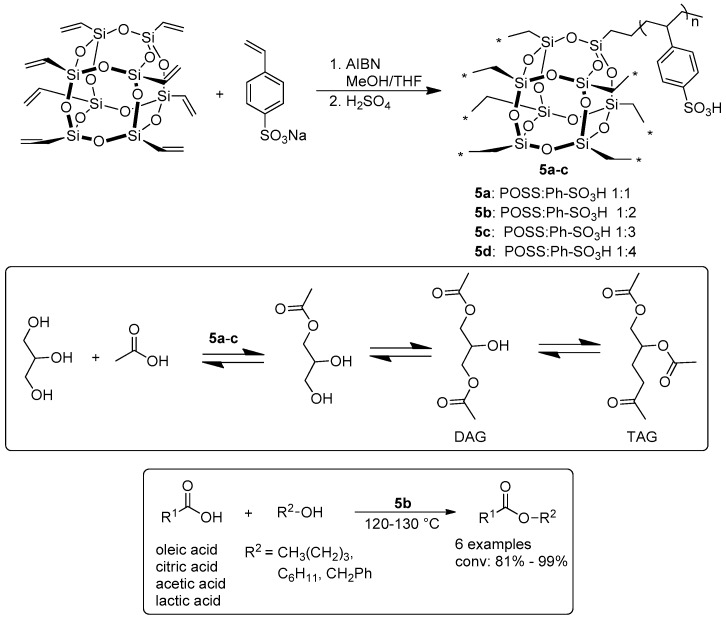
Synthesis of POSS-based poly(styrenesulphonic acid) catalysts **5a**–**c** and their use in esterification.

The same year, poly(styrene sulphonic acid) was anchored onto polyhedral oligomeric octavinyl-silsesquioxanes (o-POSS) [6], thus providing novel hydrophobic POSS-derived solid acid catalysts for esterification of glycerol, an unavoidable side product of the biodiesel production process. These POSS-derived solid acid catalysts, denoted as **5a**–**d** (Scheme 3), were prepared through two synthetic steps: (a) free radical copolymerization of octavinylsilsesquioxanes [7,8] with sodium *p*-styrene sulfonate varying their molar ratio and (b) treatment of the obtained copolymers with H_2_SO_4_. POSS-derived solid acid catalysts **5a**, **5b**, **5c**, **5d** were formed with molar ratios of POSS to Ph-SO_3_H of approximately 1:1, 1:2, 1:3, and 1:4, respectively. All synthesized catalysts presented high conversions (above 95%) with remarkable combined selectivity (above 99%) towards glyceryl diacetate (DAG) and glyceryl triacetate (TAG) in the esterification of glycerol with acetic acid at 120 °C. After each cycle, the catalyst was separated from the reaction system by filtration and reused for five-run tests without any decrease of catalytic activity. The application of **5b** was extended to the esterification of various substrates (Scheme 3), obtaining high conversions (81%–99%).

In comparison to reported solid catalysts [9,10], the esterification of glycerol with acetic acid catalysed by **5a**–**c**, was demonstrated to be superior, considering the acetic acid molar ratio, the reaction time, and the catalyst preparation. Application of **5a**–**c** in the acetalization of glycerol also gave excellent results in terms of activity and reusability [6].

**Scheme 4:**
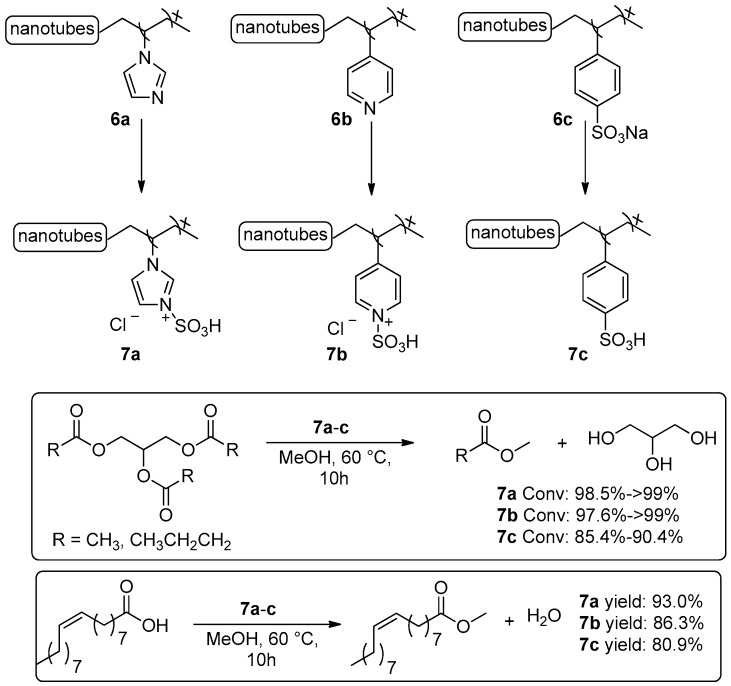
Carbon nanotube-based solid sulfonic acids **7a**–**c** as catalysts for synthesis of methyl esters via transesterification and esterification.

Recently, carbon nanotube-based solid sulfonic acids were reported as catalysts for production of fatty acid methyl esters via transesterification and esterification [11]. The investigated materials were obtained by covalent grafting of multi-walled carbon nanotubes (CNT) with sulfonic acid-functionalized polymers. In detail, polyelectrolyte brush-modified solid acid **7a** and **7b** were prepared by sulfonation of poly(1-vinylimidazole)-grafted multi-walled CNT **6a** and poly(4-vinylpyridine)-grafted multi-walled CNT **6b**, respectively, with chlorosulfonic acid (Scheme 4). The precursors **6a** and **6b** were synthesized by an in situ radical polymerization of 1-vinylimidazole and 4-vinylpyridine monomer, respectively, in the presence of multi-walled CNTs. The polyelectrolyte brush-modified solid acid **7c** was obtained by acidification of poly(sodium 4-styrenesulfonate)-grafted CNT **6c**, prepared by an in situ polymerization of sodium *p*-styrenesulfonate monomer in the presence of multi-walled CNTs. At first, the resulting catalytic materials were tested in transesterification of glyceryl triacetate and glyceryl tributyrate with methanol. All of the solid sulfonic acids **7a**–**c** showed excellent catalytic performance giving high conversions after 10 and 5 h. The methanolysis of glyceryl triacetate was proved to be much faster than the methanolysis of glyceryl tributyrate. In both reactions, the activity of catalysts **7a**–**c** decreased in the following order **7a** > **7b** > **7c**. In addition to the transesterification of triglycerides, catalysts **7a**–**c** were also effective for esterification of C18 mono-unsaturated oleic acid with methanol giving excellent yields of methyl oleate. The reusability of catalyst **7a** was investigated by transesterification of glyceryl tributyrate with methanol at a low glyceryl tributyrate conversion level. The conversion of glyceryl tributyrate decreased from 52.4% to 40.9% after six cycles. The elemental analysis of recovered catalyst revealed a decrease of –SO_3_H concentration compared to fresh catalyst. Therefore, the loss of activity of catalyst was presumably due to partial cleavage of polymeric chains covalently linked on the CNTs, which resulted in a partial loss of catalytic active sites (–SO_3_H groups).

#### 2.1.2. Beckmann Reactions

**Scheme 5:**
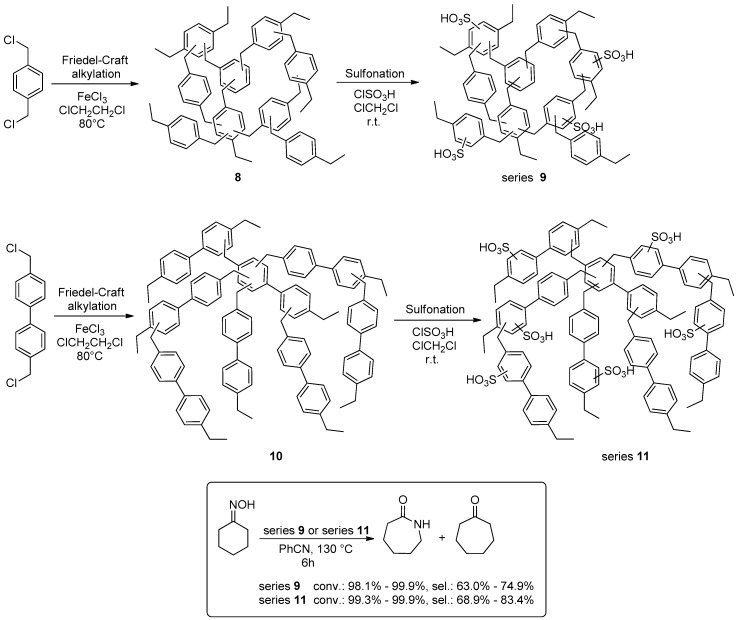
Synthesis of sulfonic acid-functionalized hyper-crosslinked polymers, series **7** and **9**, and their use in the Beckmann rearrangement of cyclohexanone oxime.

Two series of sulfonic acid-functionalized porous hyper-crosslinked polymers, denoted as series **9** and series **11**, were synthesized in two synthetic steps (Scheme 5) [12]. The first step was the self-condensation of the rigid bischloromethyl monomers, *α,α*′-dichloro-*p*-xylene (for the series **9**) or 4,4′-bis(chloromethyl)-1,1′-biphenyl (for series **11**), via FeCl_3_-induced Friedel-Crafts alkylation. The second step consisted in the treatment of each material **8** or **10** synthesized in the first step, with a variable amount of ClSO_3_H (2, 4, 8, 16 mL per gram of porous hypercrosslinked polymer) thus obtaining the two series of materials **9** or **11**, respectively. All the SO_3_H-functionalized solid acids presented high conversions (98.1%–99.9%) in the Beckmann rearrangement of cyclohexanone oxime in benzonitrile at 130 °C after 6 h.

The series **11** demonstrated superior selectivity. The highest selectivity (83.4%) was found with the sample of series **11** obtained by sulfonation with 4 mL of ClSO_3_H. The authors attributed the good catalytic performance of the **9** and **11** materials to their high acidic density, narrow dispersed pores size in the junction of micro and mesopores, large surface area of the polymeric framework and a special nano-confinement effect that was beneficial in promoting catalytic activities. In the case of small pore size, only a limited amount of substrate molecules such as cyclohexanone oxime could be accommodated within the inner pore space, promoting the efficient collision of the substrate with the acidic sites on the pore wall, which thereby accelerated the reaction.

**Scheme 6:**
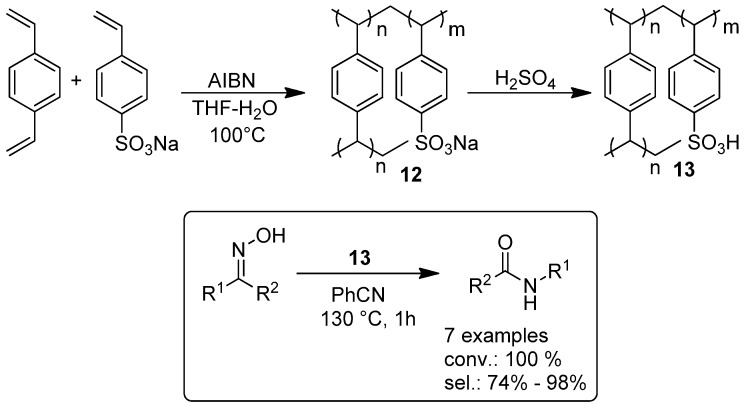
Synthesis of sulfonic acid-functionalized polymer **13** and its use as catalyst in Beckmann rearrangement.

Recently, Li et al. reported the first example of heterogeneous Beckmann reaction catalysed by non-post sulfonated polymer material [13]. Particularly, polymer **13** (Scheme 6) was prepared by acidification of polymer **12** obtained by copolymerization of divinylbenzene and sodium *p*-styrene sulfonate [14]. The sulfonic acid group-functionalized polymer **13** was tested in the Beckmann rearrangement of cyclohexanone oxime; a conversion of 100% with relatively high selectivity of 75% was obtained after 1 h at 130 °C in PhCN. High conversions and selectivities were also observed for other ketoximes, showing high efficiency and good substrate compatibility. Recyclability in the Beckmann rearrangement of cyclohexanone oxime was studied. The catalyst **13** was easily recovered by centrifugation and reused in the next run. However, a significant decrease of the conversion (conversion of only 20%) was observed. Good reusability was achieved by regenerating the recovered catalyst with dilute H_2_SO_4_. With this simple treatment, the catalyst showed superior reusability and displayed a considerably high activity, even after five recycling runs.

### 2.2. Synthesis of Cyclic Carbonates

**Scheme 7:**
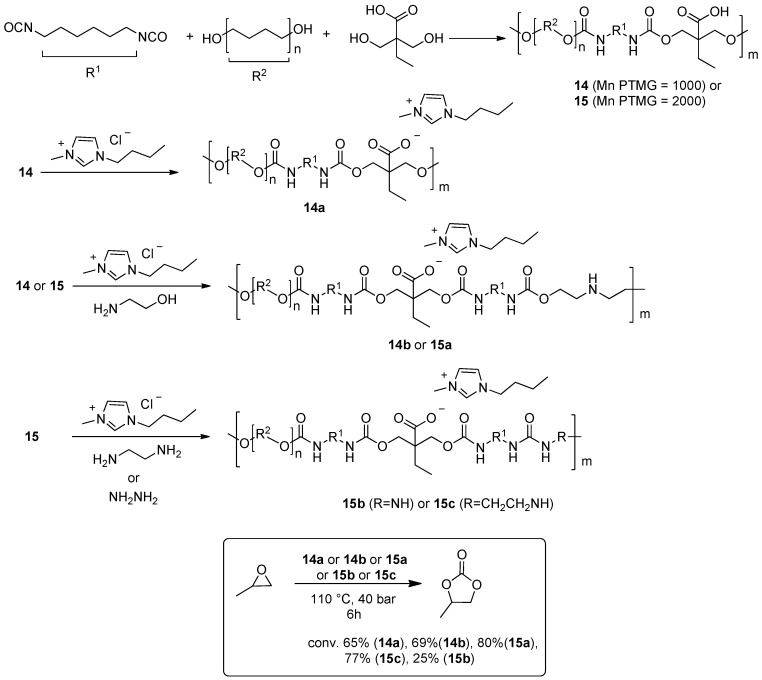
Urethane-based poly(ionic liquid)s as catalysts **14a**–**b** and **15a**–**c** in the cycloaddition of CO_2_ to propylene oxide.

Rojas et al. reported the synthesis of a series of urethane-based poly(ionic liquid)s (PILs) and their application as catalysts in the cycloaddition of CO_2_ to propylene oxide [15]. Urethane-based PILs were synthesized by addition of hexamethylenediisocyanate to dimethylolbutanoic acid and poly-(tetramethylene ether) glycol (PTMG Mn = 1000 or 2000 g/mol), and subsequent exchange reaction with 1-butyl-3-methylimidazolium chloride (Scheme 7). In addition to **14a**, without chain extension, different urethane-based PILs with chain extension **14b**, **15a**, **15b** and **15c** were prepared by adding diethanolamine (for **14b** and **15a**), hydrazine (for **15b**) and ethylenediamine (for **15c**) as chain extenders, and by varying the molecular weight of the polyol. Variation of compositions of PILs influenced their thermal and physical properties as well as their catalytic activity. The PILs with superior Mn (PTMG 2000) and higher chain extender length (**15a** and **15c**) exhibited higher activity. In fact, the chain extension improved the mobility of the PILs favouring the diffusion and solubility of CO_2_ in material, thus increasing its interaction with the active sites. These results were similar to those obtained with 1-butyl-3-methylimidazolium chloride in the same conditions (conversion 84%).The authors proposed a possible mechanism that clarified the role of the imidazolium cation and the nucleophilic group COO^−^. After each reaction, the urethane-based PILs were recovered and reused up to five times without a significant loss of its catalytic activity.

**Scheme 8:**
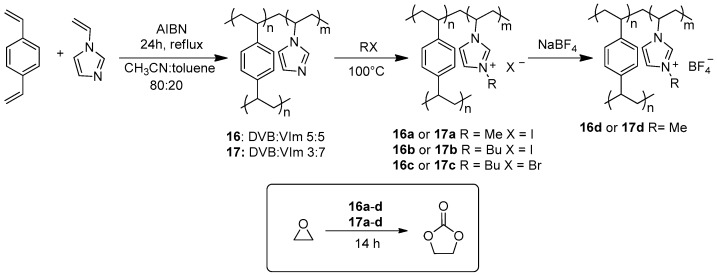
Synthesis of poly(ionic liquid)s **16a**–**d** and **17a**–**d** and their use in the formation of ethylene carbonate.

Dani et al. developed a systematic approach to understand the relation between structure of porous PILs and their properties, by testing a set of porous PILs as catalysts in the coupling reaction between CO_2_ and ethylene oxide under very mild conditions (room temperature and low pressure) by means of in-situ FTIR spectroscopy within 14 h of reaction [16]. In this regard, the series of porous PILs, **16a**–**d** and **17a**–**d** (Scheme 8), were prepared through alkylation of non-ionic co-polymers **16**–**17** synthesized by a precipitation polymerization method using vinylimidazole (VIm) as functional monomer and DVB as cross-linking agent. Polymers **16** and **17** were obtained at volume ratio of DVB:VIm of 5:5 and 3:7, respectively. The effect of the counter anion (I^−^, Br^−^ and BF_4_^−^), the length of the alkyl chain (methyl and butyl) and the cross-link degree were investigated. The catalytic performances of PILs depended on the presence of a sufficient surface area and were proportional to the VIm content. The catalyst activity increased with increasing the nucleophilicity of the anion (I^−^ > Br^−^) and length of alkyl chains, that stabilized the imidazole cation and reduced the electrostatic attraction between the ion-pair, so that the anion was more available to catalyse the reaction. PILs with BF_4_^−^ showed no activity due to the low nucleophilicity of BF_4_^−^ anion, whose high delocalized charge was not sufficient to allow the ring opening of ethylene oxide. Non-ionic co-polymers **16**–**17** showed very low activity. Finally, the catalytic activity of Poly(mVIm^+^I^−^) was very low, despite the higher concentration of active sites, as a consequence of the absence of porosity and then of the low accessibility to the active sites.

**Scheme 9:**
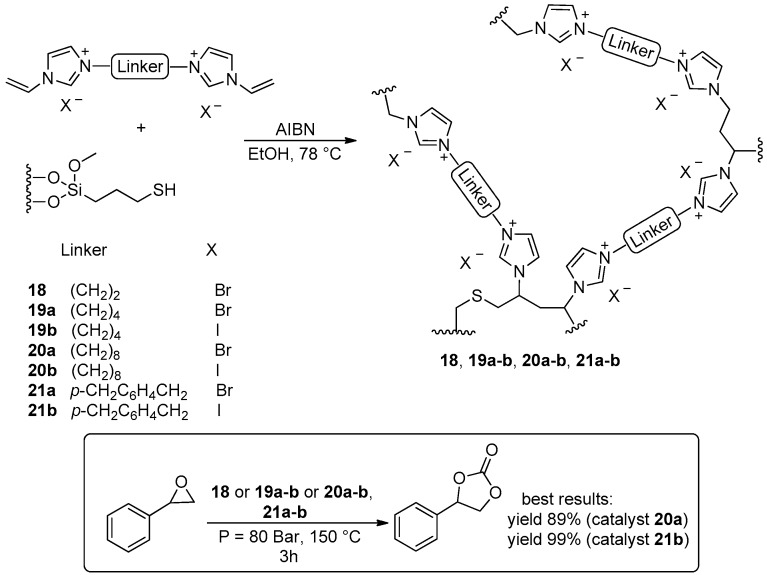
Preparation of mlc-SILP materials **18**, **19a**–**b**, **20a**–**b**, **21a**–**b** and their use as catalysts in the synthesis of styrene carbonate.

In 2014, a series of multilayered covalently-supported ionic liquid phase (mlc-SILP) materials **18**, **19a**–**b**, **20a**–**b**, **21a**–**b** were synthesised by grafting different bis-vinylimidazolium salts on thiol-functionalised silica (Scheme 9) [17]. Covalently-supported imidazolium salts represent an interesting class of recyclable catalysts with excellent stability and durability [18,19]. Bis-imidazolium salts with different organic linkers (ethane, butane, octane, *p*-xylene) between the two imidazolium units and two types of halides as counterions (X = Br or I) were used. Since the materials were synthesized using an excess of bis-imidazolium salts with respect to the SH groups of thiol-functionalized silica, a cross-linked oligomeric network of imidazolium units on the silica support was obtained. The catalytic activity of materials **18**, **19a**–**b**, **20a**–**b**, **21a**–**b** was valued and compared in the coupling reaction between CO_2_, under supercritical conditions, and two different epoxides, an aliphatic terminal epoxide (1,2-epoxyhexane) and an aromatic terminal epoxide (styrene oxide). With all catalysts, higher epoxide conversions were observed with styrene oxide as compared to 1,2-epoxy-hexane.

The materials **20b** and **21b**, having octane and *p*-xylene as linker, respectively, and iodide as counterion, were identified as the most active catalysts. These results indicated that the leaving ability of the halide played a role in determining the reaction rate and that a better separation between the imidazolium units, obtained with the linker with the longest chain (octane), could prove favourable for simultaneous reaction at both of them. The results with these new catalysts were markedly superior compared to the previous optimum mlc-SILP catalyst (SiO_2_–*p*-xylene–Br) [20]. Material **20b** was also employed in the synthesis of cyclic carbonates from different epoxides and CO_2_. In this case, higher carbonate yields were observed with the terminal epoxides, while cyclohexene oxide and cyclopentene oxide showed lower reactivity, leading to only low carbonate yields.

**Scheme 10:**
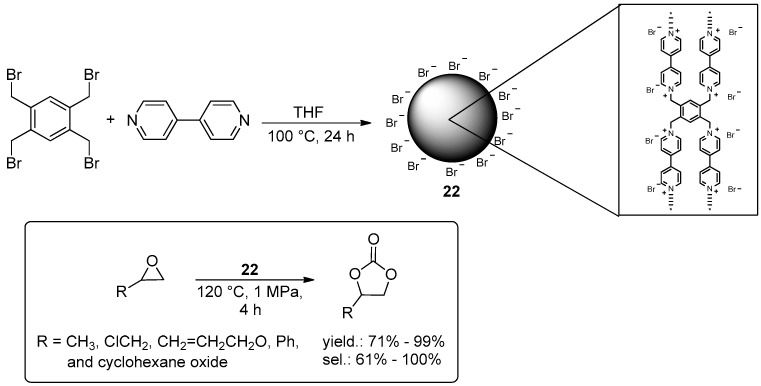
Synthesis of poly(ionic liquid) **22** and its application in the formation of cyclic carbonates.

Recently, a new type of stable highly cross-linked cationic polymer microspheres, denoted as **22**, was solvothermally synthesized from the reaction of 1,2,4,5-tetrakis(bromomethyl)benzene and 4,4′-bipyridine (Scheme 10) [21]. Various solvents, including THF, toluene, acetonitrile, and ethyl acetate were applied. The spherical particles **22** (with sizes of approximately 2–3 nm) formed in THF were the most stable and exhibited high activity and selectivity in the coupling reaction between CO_2_ and different substituted epoxides at 120 °C under metal-free and solvent-free conditions. In addition, spherical particles **22**, insoluble in the reaction system, had good recyclability, which was mainly attributed to the cross-linked covalent cationic structure. After the reaction, the cross-linked cationic polymer microspheres **22** could be easily recycled by filtration or centrifugation and reused six successive times without a significant decrease in the efficiency or structural deterioration.

**Scheme 11:**
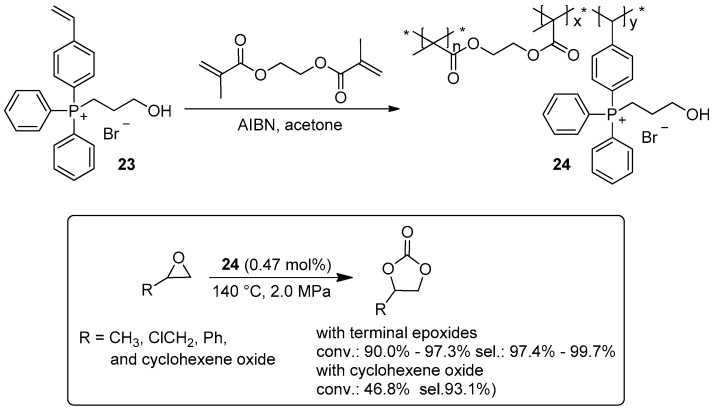
Preparation of phosphonium-based poly(ionic liquid) **24** and its use as catalyst in the synthesis of cyclic carbonates.

Among ionic liquids used as catalysts for the cycloaddition of CO_2_, the quaternary phosphonium-based ionic liquids have not been extensively studied due to their less tailored structures, limited heterogenizing method and required harsh reaction conditions. In 2014, phosphonium-based ionic liquids functionalized with hydroxyl and carboxyl groups showed good catalytic activity in the synthesis of cyclic carbonates in the absence of co-catalyst and solvent [22]. In light of the superiority of the functionalized phosphonium-based ionic liquids, recently polymer nanoparticles grafted with hydroxyl-functionalized phosphonium-based ionic liquid **24** were prepared by radical copolymerization of diphenyl(*p*-vinylphenyl)(3-hydroxylpropyl)-phosphonium bromide (**23**) with ethylene glycol dimethylacrylate (EGDMA) (Scheme 11) [23]. Under optimized conditions, the cross-linked polymer **24** exhibited good catalytic activity in the coupling reaction between CO_2_ and various terminal epoxides (yield 90.0%–97.3%). Cyclohexene oxide gave relatively low activity (46.8%), due to large steric hindrance. The catalytic activity was comparable to that of homogeneous catalyst **23** (99.5% of propylene carbonate) and was higher than homopolymers synthesized using **23** and EGDMA as monomers. Besides, **24** exhibited superior catalytic performance than polymer nanoparticles synthesized by copolymerization of triphenylpropylphosphonium bromide with EGDMA, showing the important role of hydroxyl groups. The catalyst had a good thermal stability and excellent reusability up to six cycles without any significant loss of activity.

**Scheme 12:**
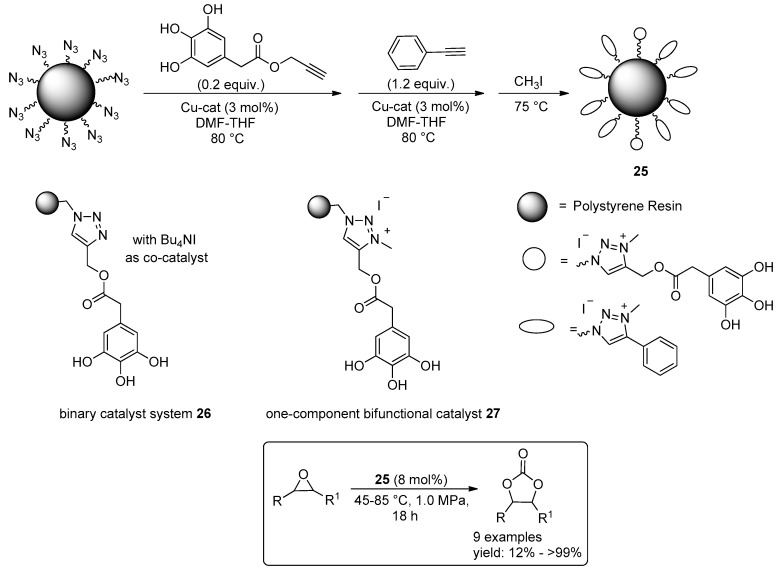
Preparation of bifunctional resin **25** and its use as catalyst in the synthesis of cyclic carbonates.

Most immobilized organocatalyst systems for the conversion of epoxides to cyclic carbonates, require operating temperatures above 100 °C, which challenges their overall sustainability. The following example reports a recyclable catalyst system that promotes the conversion of CO_2_ under mild conditions. The bifunctional catalyst **25**, where the triazolium and pyrogallol units were linked to distinct positions along the polymer backbone, was prepared starting from azidomethyl polystyrene and tested as catalyst in coupling reaction between CO_2_ and 1,2-epoxyhexane at 45 °C (Scheme 12) [24].

This bifunctional resin **25** maintained its activity level for a longer period than binary catalyst system **26** and the one-component bifunctional catalyst **27**. An appreciable reduction in activity only started to occur after five runs, but a regenerative treatment with methyl iodide permitted the recovery of catalytic activity; for example, the resin **25** was used in 11 consecutive runs giving a total of 938 turnovers/1,2,3-trihydroxybenzene unit which was significantly higher than those that might be obtained with **26** or **27**. The bifunctional catalyst **25** was employed in the synthesis of other cyclic carbonates. High yields were obtained with terminal epoxides and low yields with internal epoxides. Among internal epoxides, only conversion of indene oxide proceeded smoothly (yield > 99%).

**Scheme 13:**
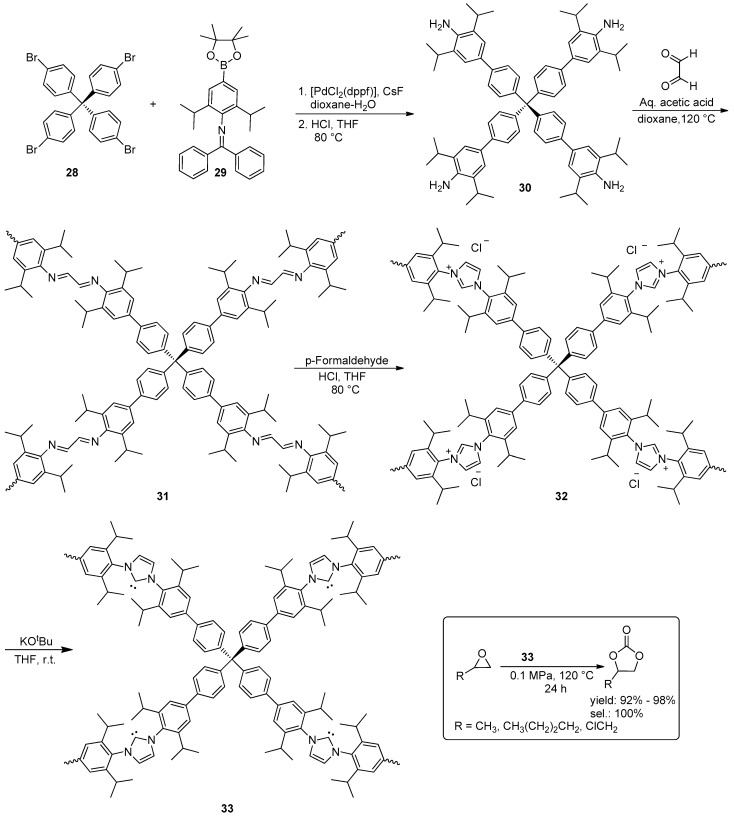
Preparation of NHC-nanoporous polymer **33** and its application in the synthesis of cyclic carbonates.

A new class of nanoporous polymers incorporating sterically-confined *N*-heterocyclic carbenes (NP–NHCs), as heterogeneous nanoporous organocatalysts for the conversion of CO_2_ into cyclic carbonates at atmospheric pressure, was reported [25]. Particularly, the nanoporous polymer NP–NHC **33** was prepared following the synthetic strategy depicted in Scheme 13.

At first, a tetrahedral core based on tetraphenyl methane incorporating 2,6-diisopropyl aniline moieties at the terminal positions, denoted as **30**, was synthesized by reaction between tetrakis(4-bromophenyl)methane (**28**) and borane **29**. After, three synthetic steps led to the polymer **33**: (a) copolymerization of **30** with glyoxal in order to obtain the polymer **31**; (b) reaction with paraformaldehyde in the presence of HCl to form **32** having the corresponding imidazolium chloride moieties and (c) generation of sterically-confined *N*-heterocyclic carbenes **33** by neutralization of the imidazolium chloride moieties with potassium *tert*-butoxide in THF. The catalytic activity of the NP–NHC polymer **33** was tested in the conversion of CO_2_ into cyclic carbonates under atmospheric pressure of CO_2_ (0.1 MPa) at 120 °C with 5 wt % catalyst loading. Excellent yields (92%–98%) and 100% product selectivity were obtained with mono aliphatic substituted terminal epoxides. Traces or no conversion for the phenyl and benzyl substituted epoxides were observed. According to the authors, this could be due to the molecular sieving property of polymer **33**, conferred by the pore diameter of polymer (~0.4 nm), which was less than that of kinetic diameter of the aromatic epoxides. In addition, the catalyst was recycled four times without any change in activity and product yields.

### 2.3. C–C Bond Forming Reactions

**Scheme 14:**
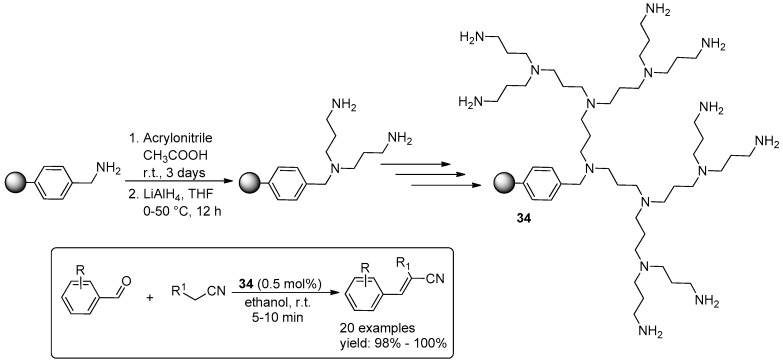
Preparation of polystyrene-supported poly(propylene imine) dendrimer **34** and its application as catalyst in Knoevenagel condensation.

In 2014, the solid phase synthesis of poly(propylene imine) (PPI) dendrimers was described for first time [26]. Particularly, polymer-supported PPI dendrimer **34** was prepared by synthesizing poly(propylene imine) dendrimer up to third generation on crosslinked polystyrene. An iterative method, which included a double Michael addition of acrylonitrile to the amino groups of the polymer support and subsequent LiAlH_4_ reduction of the nitrile groups to amino groups, was followed (Scheme 14). Material **34** was employed as heterogeneous catalyst in Knoevenagel condensations. Using only 0.5 mol % of catalyst **34**, α,β-unsaturated nitriles were obtained in excellent yield (98%–100%) in ethanol at room temperature. The yields of the products obtained with this catalyst were similar to those obtained with polystyrene-supported PAMAM dendrimers [27] in the same conditions but with the catalyst **34** the reaction time was shorter. The catalyst **34** was recovered by simple filtration and reused up to eight times without loss of catalytic activity.

**Scheme 15:**
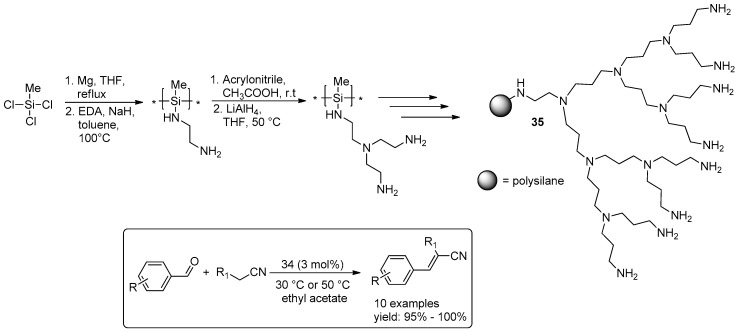
Polysilane-supported (polypropyleneimine) dendrimer **35** and its catalytic activity in Knoevenagel condensation.

The following year, polysilane-supported poly(propyleneimine) dendrimer **35** was used as catalyst in Knoevenagel condensations, showing the first example of PPI dendrimer grafted polysilane catalyst (Scheme 15) [28]. The material **35** was prepared by synthesizing the third-generation PPI dendrimer on the aminoethylpolysilane according to the general strategy of solid phase synthesis. The aminoethylpolysilane was previously prepared by condensation of trichoromethylsilane through Grignard coupling followed by the conversion of chloride groups in aminoethyl moieties with excess of ethylenediamine (EDA). Using 3 mol % of catalyst **35**, α,β-unsaturated nitriles were obtained in high yield carrying out the reaction at 30 °C and at 50 °C. Recycling of catalyst **35** was studied in the Knoevenagel condensation between benzaldehyde and malononitrile. Four cycles were carried out and the yields gradually decreased from 100% to 92%.

**Scheme 16:**
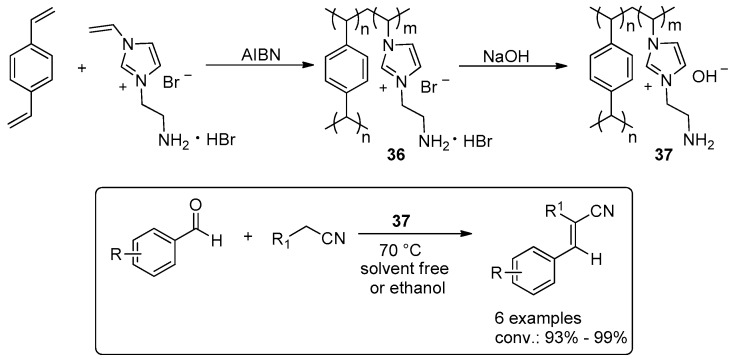
Preparation of poly(ionic liquid) **37** and its application in Knoevenagel condensation.

A new hydrophobic mesoporous poly(ionic liquid) was used as a recyclable solid-base catalyst for solvent-free Knoevenagel condensations [29]. This material, denoted as **37**, was prepared in two synthetic steps: (a) synthesis of polymer **36** by radical copolymerization of 1-aminoethyl-3-vinylimidazolium bromide hydrobromide with divinylbenzene varying the molar ratio of two monomers; (b) ion-exchange reaction with a NaOH solution (Scheme 16). Among the various molar ratios tested in the first step, the molar ratio 1:1 was optimal. In fact, the mesoporous poly(ionic liquid) **37** with equimolar amounts of divinylbenzene and ionic liquid precursor exhibited the best catalytic activity (conversion 99%) in solvent-free Knoevenagel condensation between benzaldehyde and ethyl cyanoacetate at 70 °C. It was also tested in Knoevenagel condensation with various substrates, giving high conversions (93%–99%) at 70 °C in a short reaction time. The authors proposed a possible synergistic Lewis–Brønsted dual-base-center mechanism that justified high catalytic activity. The material **37** was used for six cycles without significant loss of activity.

**Scheme 17:**
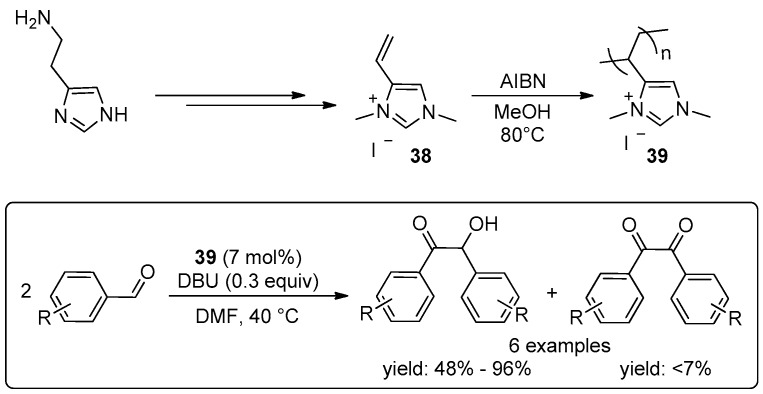
Synthesis of poly(ionic liquid) **39** and its use as catalyst in benzoin condensation.

In 2014, Seo and Chung reported the first successfully recyclable catalytic system for benzoin condensation [30]. Particularly, poly(4-vinylimidazolium) iodide [31] **39** was prepared by polymerization of 4-vinylimidazolium **38**, synthesized starting from histamine. Subsequently, the polymer was tested as an organic pre-catalyst in benzoin condensations (Scheme 17). Under optimized conditions, diverse functionalized benzoin products were obtained in 48%–96% yield with benzyl as by-product in various yields (less than 7%). In all cases, pre-catalyst **39** exhibited higher catalytic activity than the corresponding monomeric analogue **38**. Moreover, **39** showed higher catalytic activity and reusability in the benzoin condensation reaction compared to the polymerized pre-catalysts obtained from 1-vinylimidazolium [32,33]. The pre-catalyst **39** was recovered and reused without loss of performance over seven cycles. The material **39** showed high catalytic activity even in the tandem formation of γ-butyrolactone from benzaldehyde and methyl acrylate [31].

**Scheme 18:**
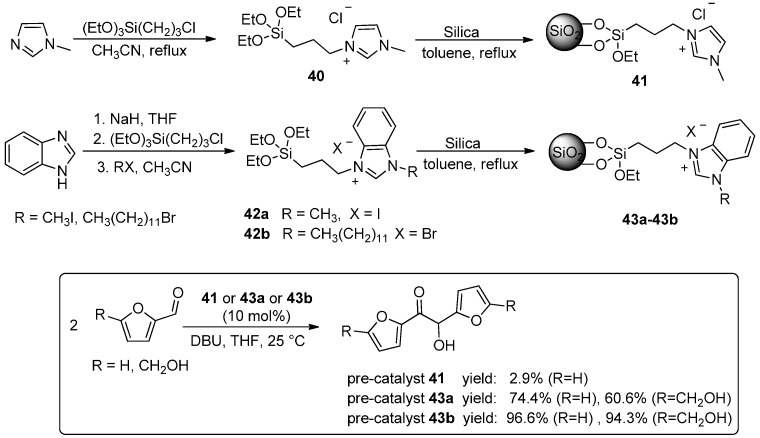
Preparation of silica-supported ionic-liquids **41**, **43a**–**b** and their application as catalysts in the synthesis of furoins.

In 2015 Wang and Chen reported the first highly efficient and fully recyclable heterogeneous catalyst systems for self-coupling of furaldehydes, particularly furfural and 5-hydroxymethylfurfural [34]. At first, three pre-catalyst structures **41**, **43a**, **43b** were prepared by grafting of the respective triethoxysilylpropyl functionalized azolium salts **44**, **46a**, **46b** onto the silica surface (Scheme 18). The catalytic performances of **41**, **43a**, **43b** were investigated in self-coupling reactions of furfural and 5-hydroxymethylfurfural into their respective furoins.

**Scheme 19:**
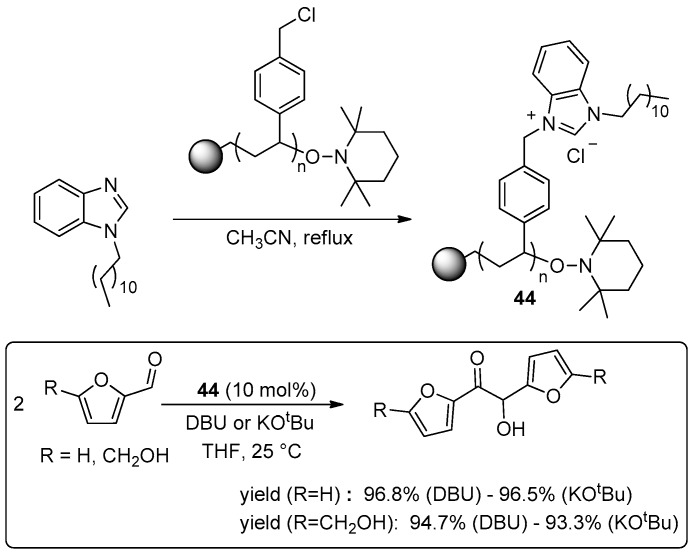
Preparation of poly(ionic liquid) **44** and its application in the synthesis of furoins.

Benzimidazolium pre-catalyst **43b**, carrying a long-chain (C12) substituent, showed the best catalytic activity, producing furoins with high yields (94.3%–96.6%). According to the authors, the higher activity of **43b** could be attributed to presence of sterically demanding substituents, that facilitated the shift of the Wanzlick equilibrium (equilibrium between the carbene and its corresponding dimer) toward the formation of free carbenes [35]. The long-chain alkyl group placed in the proximity of the carbene center could also provide a hydrophobic protection pocket for the catalyst to minimize its decomposition or poisoning caused by adventitious protic or oxidative sources. After this preliminary study, the Merrifield’s peptide resin, an organic support, was utilized to synthesize the grafted salt pre-catalyst **44** (Scheme 19) [34]. 1-Dodecylbenzimidazole, prepared from reaction between benzimidazolide and dodecyl bromide, was grafted onto the Merrifield’s peptide resin through alkylation of the 1-dodecylbenzimidazole with the benzyl chloride functional group contained in the resin. In presence of DBU or KO^t^Bu, the pre-catalyst **44** showed excellent catalytic activity in the self-coupling reaction of furfural (96.8% yield with DBU and 96.5% with KO^t^Bu) and in the self-coupling reaction of 5-hydroxymethylfurfural (94.7% with DBU and 93.3% with KO^t^Bu). Both catalytic systems **43b** and **44** showed excellent recyclability in furfural self-coupling without loss of the catalytic activity after ten cycles. This result was obtained by adopting a catalyst recycling procedure that involves activation of the pre-catalyst with a base to generate the NHC catalyst and recycle of the catalyst by quenching the reaction with HCl to convert the catalyst back to the pre-catalyst.

### 2.4. Miscellaneous Examples

**Scheme 20:**
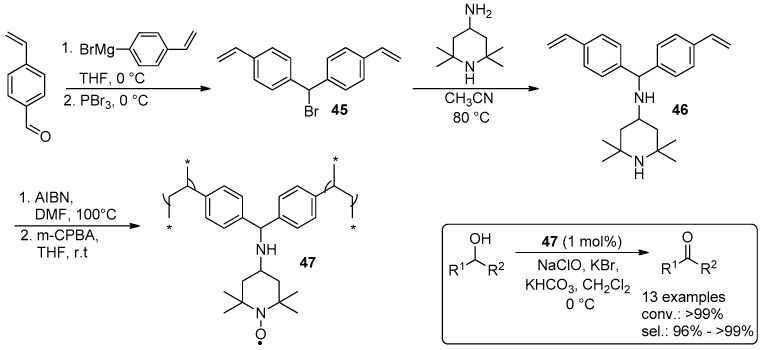
Preparation of porous polymerized organocatalyst **47** and its application in the oxidation of alcohols.

A new porous polymerized organocatalyst with high surface area, hierarchical porosity, and superior chemical stability was prepared and employed as an efficient heterogeneous catalyst in the oxidation of alcohols. In detail, the porous polymerized organocatalyst **47** was synthesized from solvothermal polymerization of vinyl-functionalized 2,2,6,6-tetramethylpiperidine monomer **46** in the presence of AIBN at 100 °C, followed by oxidation with *m*-chloroperbenzoic acid (*m*-CPBA) (Scheme 20) [36]. Monomer **46** was synthesized by nucleophilic substitution reaction between 2,2,6,6-tetramethyl-4-piperidylamine and 4,4′-(bromomethylene)-bis(vinylbenzene) (**45**), previously prepared starting from 4-vinylbenzaldehyde. The use of 1 mol % of **47** in oxidation of various substituted alcohols at 0 °C led to the corresponding aldehydes and ketones with very high conversions (>99%) and selectivities (96%–>99%). The reaction with benzyl alcohol was also carried out with 0.1 mol % and 0.05 mol % of catalyst, obtaining a conversion of 99% after 10 and 20 h respectively. Furthermore, catalyst **47** was more active than TEMPO immobilized on MCM-41 [37]. The same conversions were achieved using polymers with superior surface area, obtained by copolymerization of monomer **46** and divinylbenzene. Catalyst **47** was used for five cycles without significant loss of activity.

**Scheme 21:**
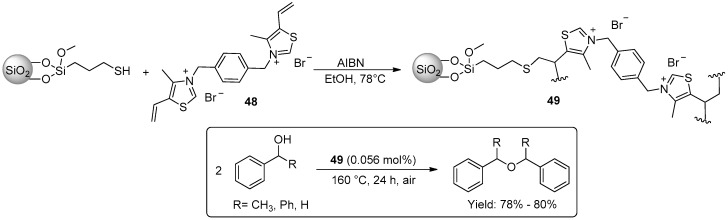
Silica-immobilized polythiazolium network **49** and its activity in etherification.

The first application of thiazolium-based compounds as catalysts for the etherification reaction of alcohols was reported [38]. Thiazolium hybrid material **49** was prepared by a radical reaction between a mercaptopropyl-modified SBA-15 mesoporous silica and large excess of bis-vinyl-thiazolium salt **48** (Scheme 21) and successively employed in the etherification reaction of 1-phenyl-ethanol under solvent-free conditions at 160 °C testing different gas phases (oxygen, air, nitrogen and argon). After 24 h, an almost quantitative conversion (93%) was observed in both oxygen and air with a better selectivity toward ether in air (86%) than oxygen (73%). Under a nitrogen or argon atmosphere, a decrease of conversion (55%, 57% respectively) and an increase of selectivity (93%, 91% respectively) were observed. Furthermore, thiazolium hybrid material **49** displayed higher conversion and selectivity compared to the analogous imidazolium-based material, thus showing that the nature of the *N-*heterocyclic ring had a determinant impact on the performances of the reaction.

The authors proposed a possible reaction mechanism. The supported thiazolium-based material **49** was also used with success in the etherification reaction of benzyl alcohol and diphenylmethanol under air. Recycle experiments were carried out in the etherification reaction of 1-phenylethanol; excellent catalytic performances were preserved in seven catalytic runs.

**Scheme 22:**
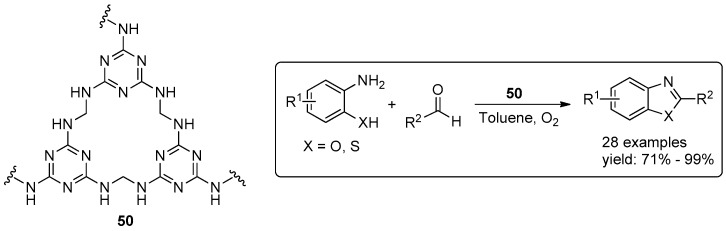
Mesoporous poly(melamine–formaldehyde) **50** as catalyst for the synthesis of benzoxazoles and benzothiazoles.

A simple, green, and efficient strategy for the synthesis of benzoxazoles and benzothiazoles was developed [39] by using the mesoporous poly(melamine–formaldehyde) **50** [40,41,42] prepared starting from melamine and paraformaldehyde (Scheme 22). Polymer **50** acted as bifunctional catalyst and, under the optimized catalytic conditions, led to benzoxazoles and benzothiazoles with good and excellent yields (71%–99%) by reaction of substituted 2-aminophenols with benzaldehydes using dioxygen as oxidant. Traces of products were obtained using aliphatic aldehydes and 2-methyl-benzaldehydes. The authors proposed a possible reaction mechanism. The catalyst was used six cycles without significant loss of catalytic performance.

**Scheme 23:**
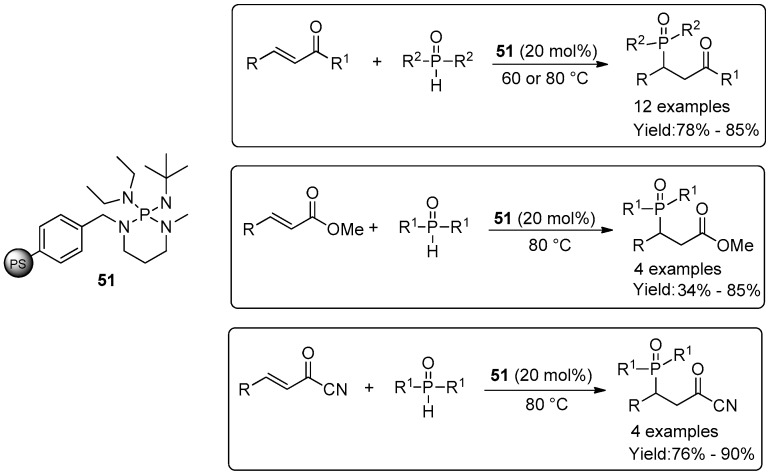
Synthesis of diethyl and dimethyl phosphonates catalysed by (PS-BEMP) **51**.

Recently, Vaccaro et al. reported the first application of a supported base as catalyst for the phospha-Michael addition of phosphorus nucleophiles to a variety of electron-poor alkenes [43]. The synthesis of different types of diethyl and dimethyl phosphonates under solvent free conditions in presence of 20 mol % of dimethylperhydro-1,3,2-diazaphosphorine-supported on polystyrene (PS-BEMP) **51** [44,45] as heterogeneous catalyst was carried out (Scheme 23). Good yields (78%–85%) were obtained using two phosphorus nucleophiles, diethyl phosphite and dimethyl phosphite, and a wide array of aromatic and non-aromatic α,β-unsaturated ketones as electrophiles.

This protocol was also extended to α,β-unsaturated esters and nitriles. Good yields were obtained with methyl acrylate (79%–85%), less satisfactory results with methyl crotonate (34%–52%). Excellent results were obtained with the α,β-unsaturated nitriles, acrylonitrile and cinnamonitrile (yields: 76%–90%). Catalyst **51** was not recyclable: all attempts to reuse the recovered catalyst in consecutive reaction runs failed.

## 3. Organocatalysed Asymmetric Reactions

Organocatalysis is a useful synthetic method also applied in asymmetric reactions and polymer immobilization of chiral catalysts is still a topic of interest [46,47]. In this section, one example of asymmetric α-amination of 1,3-dicarbonyl compounds and some examples of C–C forming reactions including aza-Henry reaction, Michael addition, α-alkylation of aldehyde, Diels–Alder reaction and aldol reaction, are reported. Finally, an example of the enantioselective flow synthesis of pyranonaphthoquinones is also described. In this contest, chiral supported organocatalysts based on bifunctional thioureas, a fluorinated amine, imidazolidinones, proline derivatives and a squaramide were examined.

**Scheme 24:**
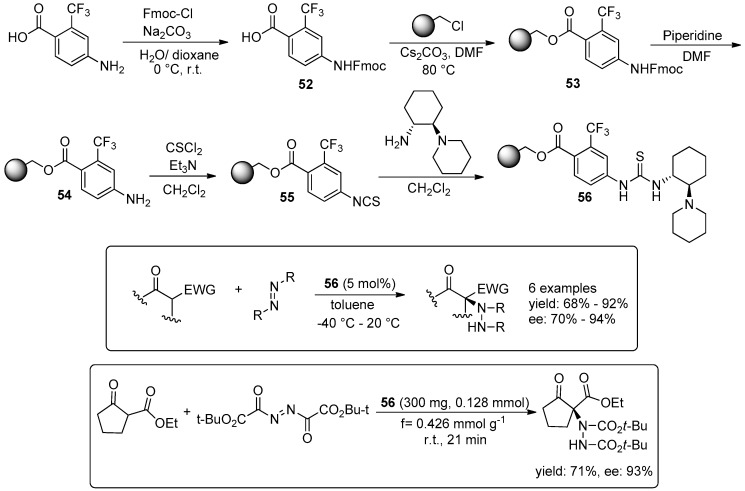
Synthesis of organocatalyst **56** and its use in the enantioselective α-amination of 1,3-dicarbonyl compounds.

In 2015, the synthesis of polymer-supported bifunctional thiourea organocatalyst **56** and its application in the enantioselective α-amination of 1,3-dicarbonyl compounds with azodicarboxylates were investigated (Scheme 24) [48]. Catalyst **56** was prepared in few steps from 4-amino-2-(trifluoro-methyl)benzoic acid avoiding the use of linkers/spacers. The Fmoc-protected amino acid **52** was immobilized onto a 1% DVB Merrifield resin, affording the material **53**, and successively was deprotected, achieving the PS-supported amino ester **54**. Then, the formation of the isothiocyanate resin **55** and subsequent reaction with enantiopure (1*R*,2*R*)-2-(piperidin-1-yl)cyclohexylamine led to polymer-supported bifunctional thiourea organocatalyst **56**. Catalyst **56** was tested in the enantioselective α-amination of a family of cyclic 1,3-dicarbonyl compounds with di-*tert*-butyl azo-dicarboxylate and diethyl azodicarboxylate. High yields and enantioselectivity values were obtained with 5 mol % of the bifunctional catalyst in toluene. Recycling experiments in the α-amination of ethyl 2-oxocyclopentanecarboxylate with diethyl azodicarboxylate were carried out. High isolated yields and ee′s were consistently recorded over nine cycles, regenerating the catalyst with triethylamine after each run. The α-amination of ethyl 2-oxocyclopentanecarboxylate with di-*tert*-butyl azodicarboxylate catalysed by resin **56** was also carried out in continuous flow. This experiment led to the desired product with 93% ee in 71% isolated yield with a productivity of 4.88 mmol_prod_ mmol_cat_^−1^·h^−1^, a TON of 37 and the residence time of 21 min.

**Scheme 25:**
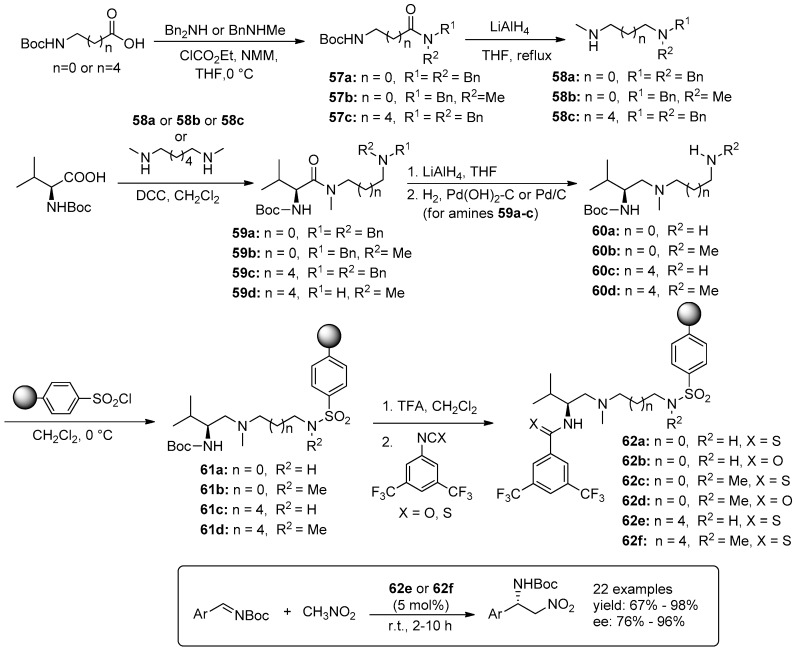
Preparation of supported organocatalysts **62a**–**f** and use of **62e**–**f** in aza-Henry reaction.

Novel bifunctional ureas and thioureas immobilized on sulfonylpolystyrene were prepared and used as recoverable and reusable organocatalysts in the stereoselective aza-Henry reaction under solvent-free conditions [49]. These catalysts (**62a**–**f**) were prepared by the synthetic strategy shown in Scheme 25. At first, the 1,2 diamines **58a**–**b** and the 1,6-diamine **58c** were synthesized starting from Boc-glycine and 6-(*N*-Boc-amino)hexanoic acid, respectively, by reaction with dibenzylamine or benzyl methylamine and successive reduction of corresponding amides **57a**–**c** with lithium aluminum hydride. Then, chiral triamines **60a**–**d** were obtained by reaction of L-valine with diamines **58a**–**c** or the commercially available 1,6-*N,N*′*-*dimethyl hexane diamine and sequential chemoselective reduction of the amide function with lithium aluminium hydride, followed by hydrogenolysis of the benzyl groups in case of amines **59a**–**c**. Chiral triamines **60a**–**d** were immobilized on sulfonylpolystyrene, affording materials **61a**–**d**. Finally, the deprotection with trifluoroacetic acid and the condensation with 3,5-bis(trifluoromethyl) phenyl isocyanate or isothiocyanate led to supported ureas and thioureas **62a**–**f**. Optimization of the reaction conditions and screening of catalysts **62a**–**f** were studied in the reaction of *N*-Boc-benzaldimine with nitromethane. The activity and enantioselectivity of the catalysts were dependent on the length of the tether connecting the urea or thiourea functionality with the polymer. The catalysts **62e**–**f**, derived from 1,6-hexane diamine, showed the best performances, in 5 mol % loading without solvent and at room temperature. Under these conditions, the catalyst **62e** and **62f** were tested in the reaction of nitromethane with different *N*-Boc aldimines. A general trend showed that the catalyst **62f** was better than **62e**, giving products in higher yields and enantioselectivities except for two imines with substituents with strong electron withdrawing effect and an imine derived from 1-naphtaldehyde. Catalysts **62e** and **62f** were recycled for three and five times, respectively, without loss of enantioselectivity and with slight decrease in the activity.

**Scheme 26:**
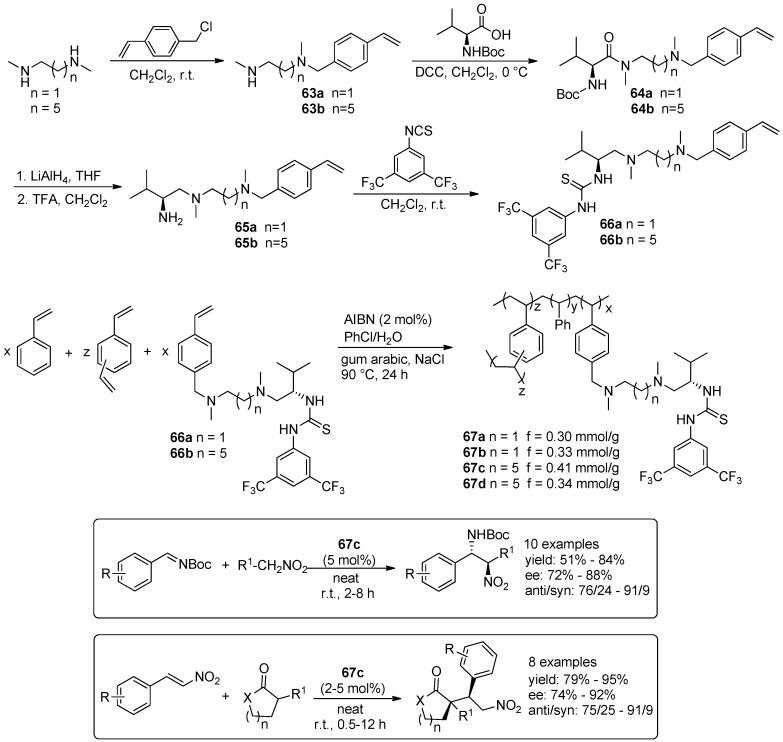
Preparation of supported thiourea-based organocatalysts **67a**–**d** and use of **67c** in aza-Henry and Michael reactions.

Recently, two sets of supported chiral thioureas, denoted as **67a**–**b** and **67c**–**d**, which differed in the length of the tether connecting the thiourea group to the polymer chain and in the effective functionalization, were prepared by copolymerization of styrene, divinylbenzene and novel styrylthiourea **66a** or **66b** respectively (Scheme 26) [50].

Styrylthioureas **66a** and **66b** were synthesized starting from commercially available *N,N′*-dimethylethylenediamine and *N,N*′-dimethyl-1,6-hexanediamine, respectively, by a common synthetic strategy which consisted in the following steps: (a) monoalkylation of previous diamines with 4-vinylbenzyl chloride to afford **63a** and **63b**; (b) reaction between Boc-L-valine and **63a** or **63b**; (c) reduction of the amide groups of **64a** and **64b** with LiAlH_4_; (d) removal of Boc protecting group by treatment with TFA; (e) reaction between 3,5-(bis)trifluoromethylisothiocyanate and triamine **65a** or **65b**.

The catalytic activity of supported chiral thioureas **67a**–**d** was tested in aza-Henry reactions between of *N*-Boc-benzaldimine and nitromethane without solvent. The catalyst **67c** was used to extend the aza-Henry reaction between different aromatic *N*-Boc-aldimines and nitroalkanes. In the presence of catalyst **67c**, moderate to good enantioselectivities (ee: 72%–88%) and yields (51%–84%) were obtained in reactions with nitromethane. The two α-nitroamines with two contiguous stereocenters, synthesized starting from nitroethane and nitropropane, were obtained with high yields (75%–84%) and enantioselectivities (ee: 88% for both examples), but with only moderate diastereoselectivity in case of the reaction with nitroethane (76:24). The catalyst **67c** was also employed in stereoselective nitro-Michael addition between various nitroolefins and nucleophiles with different cyclic structures and activating groups. Moderate to high yields (79%–95%), diastereoselectivities (75:25–91:9) and enantioselectivities (74%–92%) were obtained. The recyclability was studied for both aza-Henry reaction and nitro-Michael addition. In both cases, the catalyst **67c** showed to be recyclable maintaining the level of stereocontrol and the catalytic activity.

**Scheme 27:**
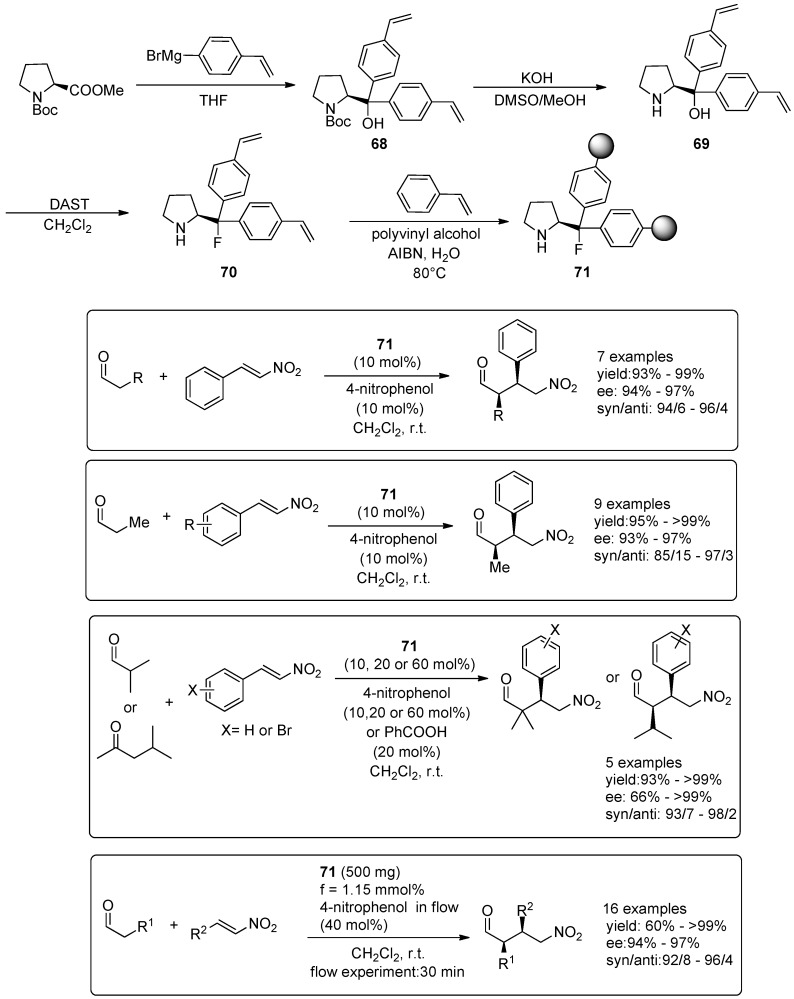
Synthesis of the polystyrene-supported fluorinated organocatalyst **71** and its application in the enantioselective Michael addition of aldehydes to nitroalkenes.

Sagamanova et al. reported the synthesis of the novel polystyrene-supported fluorinated organocatalyst **71** and its application in enantioselective Michael addition of aldehydes to nitroalkenes (Scheme 27) [51]. The supported organocatalyst **71** was prepared by co-polymerization of the chiral divinylated derivative **70** with styrene. The monomer **70** was synthesized by addition of 4-vinylphenyl magnesium bromide (prepared in situ) to the Boc-L proline methyl ester, followed by removal Boc group in basic media and deoxyfluorination of the obtained amino alcohol **69** by treatment with diethylaminosulfur trifluoride (DAST).

**Scheme 28:**
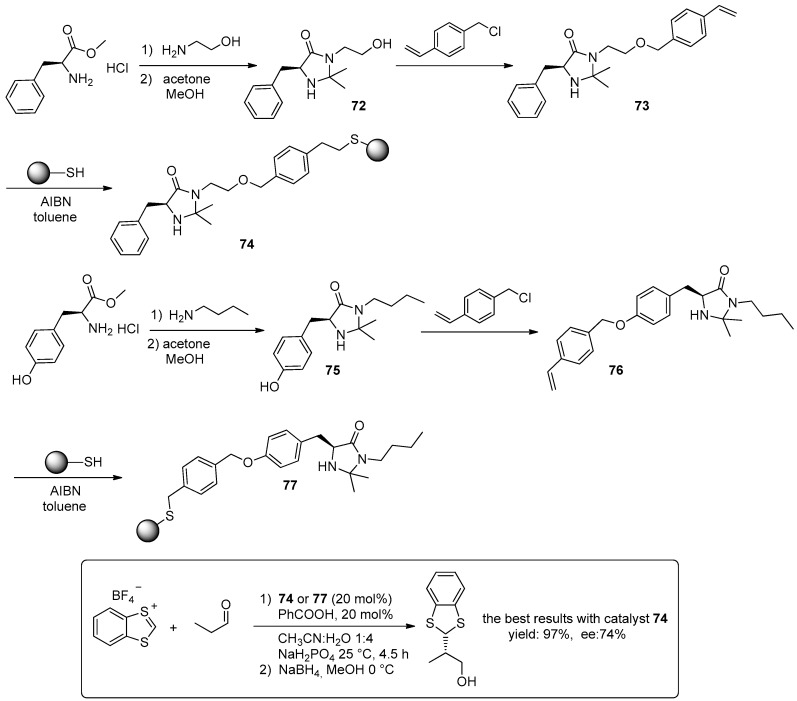
Preparation of supported organocatalysts **74** and **77** and their use in the asymmetric α-alkylation of propanal.

Effect of additives, reaction medium, loading of catalyst in addition of propanal to β-nitrostyrene were studied. The catalyst **71** (10 mol %) was employed in enantioselective Michael addition of a wide variety of aldehydes with β-nitrostyrene and in addition of propanal with different nitroalkenes under optimized conditions (10 mol % of catalyst, 10 mol % of 4-nitrophenol as additive, CH_2_Cl_2_ as solvent and room temperature). Michael adducts were obtained with excellent yields and stereoselectivities. Furthermore, high streoselectivities and yields were obtained in reaction between isovaleraldehyde or isobutyraldehyde and different nitroalkenes, varying the type of additive or loading of catalyst and additive. Recycling experiments were carried out in reaction between propanal and β-nitrostyrene. The catalyst was recycled eight times. The stereoselectivity remained constant in all runs and some progressive loss of activity was observed in the last cycles. The catalyst **71** was also applied in a carefully designed continuous flow set-up, which allowed the facile isolation of the desired Michael adducts without chromatographic purification. The multigram synthesis of a single Michael adduct over a 13 h period and the sequential generation of a library of enantiopure Michael adducts from different combinations of substrates (16 examples, 16 runs, 18.5 h total operation) were observed. The immobilized system showed comparable activity with the batch process. Two polystyrene-supported imidazolidinone catalysts, **74** and **77**, were also prepared (Scheme 28) [52].

In catalyst **74**, the chiral imidazolidinone unit was linked to polystyrene through the *N*-3 atom. In catalyst **77**, the chiral imidazolidinone unit was linked to polystyrene through the 4-position of the phenyl ring. Polystyrene-supported imidazolidinone catalysts **74** and **77** were prepared starting from L-phenylalanine methyl ester and tyrosine methyl ester, respectively, by a procedure, which included the synthesis of an amide with ethanolamine in the first case or with butylamine in the second case, followed by reaction with acetone. Both imidazolidinones thus synthesized, **72** and **75**, were modified with 4-(chloromethyl)styrene and anchored to mercaptomethyl-modified polystyrene resin by the thiol-ene addition in presence of AIBN. The catalytic activity of two polystyrene-supported catalysts **74** and **77** was compared towards the asymmetric α-alkylation of propanal with benzodithiolylium tetrafluoroborate in CH_3_CN as solvent and in presence of benzoic acid as additive. Catalyst **74** proved to be more active and enantioselective than **77**, affording the final products with yield of 97% and ee of 74% at the same levels of activity and enantioselectivity of the unsupported counterpart **73**. Catalyst **74** was highly recyclable for at least eight cycles with no loss of activity and enantioselectivity, whereas with catalyst **77** the yield and enantioselectivity decreased upon recycling.

**Scheme 29:**
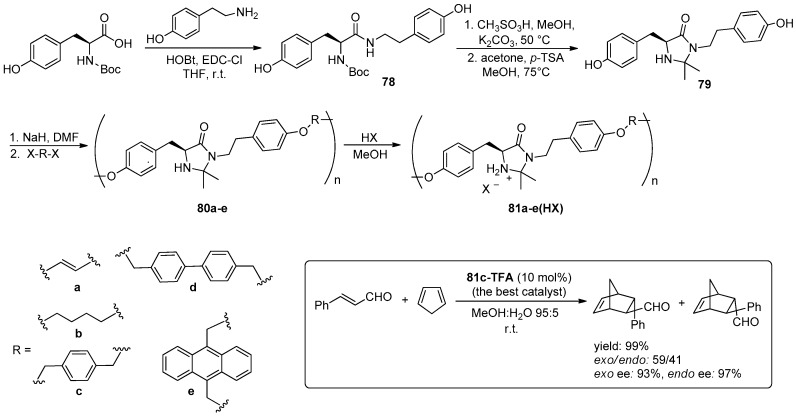
Synthesis of polyetheres **81a**–**e**(**HX**) and use of **81c-TFA** as catalyst in asymmetric Diels-Alder reaction.

Chiral polyethers, containing imidazolidinone repeating units, were synthesized and employed as catalysts in asymmetric Diels–Alder reaction [53]. These polyethers, denoted as **81a**–**e**(**HX**), were prepared following the synthetic route depicted in Scheme 29. At first, the bisphenol-type chiral imidazolidinone monomer **79** was afforded by reaction between the Boc-protected (S)-tyrosine and 4-hydroxyphenylethylamine, followed by deprotection of *N*-Boc group and reaction with acetone.

Then, the Williamson synthesis of the chiral bisphenols **79** with dihalides afforded to polyethers **80a**–**e**, whose acid treatment led to the polyethers **81a**–**e**(**HX**), having imidazolidinone salt in each repeating unit. Various types of dihalides (X–R–X) including allylic dihalide, alkyl dihalide, and benzylicdihalides and various types of acid, including HBF_4_, CF_3_COOH, CH_3_SO_3_H, TolSO_3_H, HCl, HClO_4_, were used to prepare these chiral polyethers. Effect of achiral linker R and counter anion of iminium cation on the enantioselectivity were investigated in the Diels–Alder reaction of cinnamaldehyde and cyclopentadiene. The chiral imidazolidinone polymer **81c-TFA**, having *p*-xylyl as linker and CF_3_CO_2_^−^ as counter anion, exhibited the highest enantioselectivities (*exo* ee 93%, *endo* ee 97%). After the completion of the reaction, the polymeric catalyst **81c-TFA** was recovered by simple decantation. The acid retreatment of the recovered polymeric catalyst allowed to repeatedly use the catalyst without the loss of catalytic activity.

**Scheme 30:**
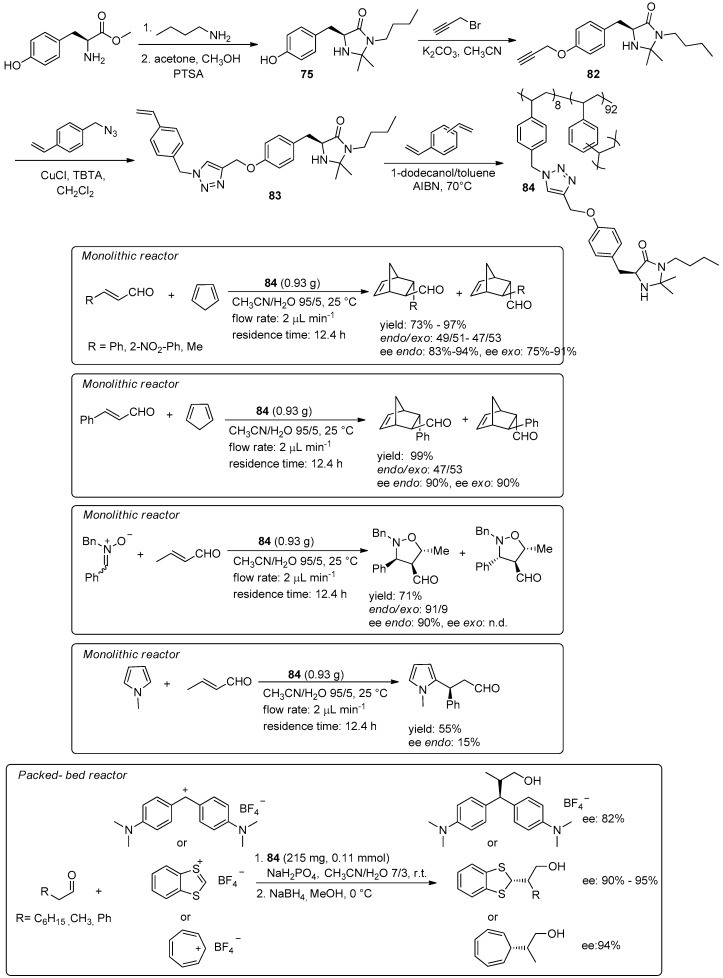
Synthesis of polymer-supported organocatalyst **84** and its application for catalysis in flow.

The first example of monolithic reactor containing a chiral organocatalyst was reported [54]. Particularly, the monolithic organocatalyst **84** was provided by radical copolymerisation of divinylbenzene and the properly modified enantiomerically pure imidazolidinone **83** inside a stainless steel column in the presence of dodecanol and toluene as porogens and AIBN as initiator (Scheme 30).

The enantiomerically pure imidazolidinone **83** was synthesized by the following synthetic strategy. The imidazolidinone **75**, synthesized starting from (*S*)-tyrosine methyl ester, was modified with propargyl bromide to afford the derivative **82** bearing a carbon-carbon triple bond. This latter was reacted with 4-(azidomethyl)styrene in the presence of CuCl and tris[(1-benzyl-1*H*-1,2,3-triazol-4-yl)methyl]amine (TBTA), thus obtaining the monomer **83**.

Diels–Alder reactions between cyclopentadiene and three different α,β-unsaturated aldehydes were carried out under continuous-flow conditions through the monolithic reactor containing the imidazolidinone organocatalyst **84** activated by treatment with HBF_4_. Diels-Alder adducts were obtained in high yield (73%–97%) and excellent enantioselectivities (ee’s: 83%–94% for *endo* diasteroisomer, ee’s: 75%–91% for *exo* diastereoisomer). Three different stereoselective transformations in continuo (Diels–Alder, 1,3-dipolar nitrone-olefin cycloaddition, and Friedel–Crafts alkylation) were also carried out in the same catalytic reactor for a total of more than 300 h on stream. Excellent results were obtained in the case of Diels–Alder and 1,3-dipolar nitrone-olefin cycloaddition (99% yield, 93% ee and 71% yield, 90% ee, at 25 °C, respectively).

The polymer-supported organocatalyst **84** was also used for preparing a packed-bed reactor, whereby the intermolecular organocatalysed enantioselective alkylation of aldehydes was performed for the first time under continuous flow conditions (Scheme 30) [55]. Excellent enantioselectivities at different flow rates were obtained in asymmetric alkylation of propionaldehyde with 1,3-benzo-dithiolylium tetrafluoroborate, tropylium tetrafluoroborate or bis[4-(dimethylamino)phenyl]-methylium tetrafluoroborate at room temperature. Excellent results were also obtained in continuous flow alkylation of phenylacetaldehyde and octanal with 1,3-benzodithiolylium tetrafluoroborate.

**Scheme 31:**
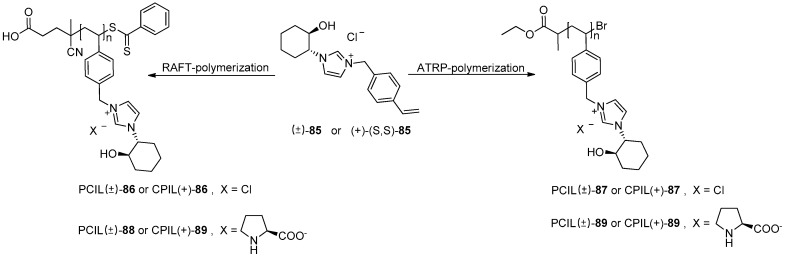
Preparation of polymeric chiral ionic liquids, PCILs-**86**-**89**.

In 2014, Karjalainen et al. reported the synthesis and application of new polymeric chiral ionic liquids (PCILs) as artificial aldolase biomimetic systems for the aldol reaction [56]. These polymers were prepared by controlled radical polymerization of monomeric units containing chiral ionic liquids (CILs). In detail, the racemic and enantiopure monomers (±)-**85** and (+)-(*S,S*)-**85** were polymerized by reversible addition–fragmentation chain transfer (RAFT) polymerization, in order to afford the corresponding polymers PCIL-(±)-**86** and PCIL-(+)-**86** and, by atom transfer radical polymerization (ATRP), in order to afford the polymers PCIL-(±)-**87** and PCIL-(+)-**87**, respectively (Scheme 31). Then, the exchange of the respective chloride counter ion with the L-prolinate anion led to new type polymeric catalysts PCIL-(±)-**88**, PCIL-(+)-**88**, PCIL-(±)-**89**, PCIL-(+)-**89**. All polymers bearing l-proline units and polymers PCIL-(+)-**86** and PCIL-(+)-**87** not bearing proline units were tested as catalysts for the aldol reaction between *p*-nitrobenzaldehyde and acetone in water. The reaction was performed with 40 mol % loading of the corresponding polymeric catalysts at room temperature for 20 h. The polymers PCIL-(+)-**86** and PCIL-(+)-**87** showed some catalytic activity (15% and 41% of conversion respectively). The polymeric derivatives bearing l-proline units consumed almost quantitatively the aldehyde but had a very poor stereoselectivity. Different amounts of the dehydrated product were also observed. These chiral polymeric catalysts were more active than the corresponding monomeric counterpart, when the reaction was carried out in water or in the presence of water.

**Scheme 32:**
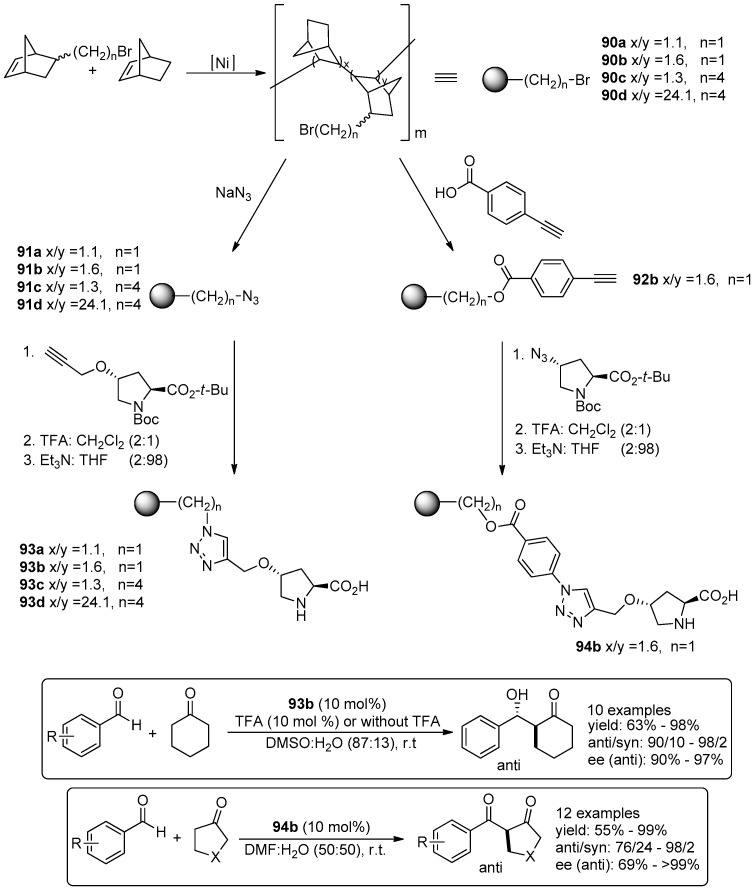
Synthesis of polynorbornene-supported proline **93a**–**d** and **94d** and their application in aldol reaction.

Sagamanova et al. reported the preparation of polynorbornene–supported proline **93a**–**d** and **94d** and their application as catalysts in the direct asymmetric aldol reaction [57]. The catalysts **93a**–**d** and **94b** were prepared following the synthetic strategy depicted in the Scheme 32. In the first step, the ω-bromoalkyl functionalized polynorbornene (**90a**–**d**), having bromoalkyl chains of different length (*n* = 1 and 4) and two levels of functionalization for each bromoalkyl chain length, were prepared by Ni–catalyzed copolymerization of norbornene and ω-bromoalkylnorbornenes [58]. Then, copolymers **90a**–**d** were treated with sodium azide to afford ω-azidoalkyl functionalized polynorbornene **91a**–**d**. Copolymer **90b** (*x*/*y* = 1.6; *n* = 1) was also treated with *p*-ethynylbenzoic acid to afford the alkynyl functionalized resin **92b**. Copolymers **91a**–**d** and **92b** were transformed into catalytic resins **93a**–**d** and **94b** through two complementary alkyne-azide click reactions followed by deprotection of *N-*Boc and t-butyl ester group with TFA in dichloromethane. Catalysts **93a**–**d**, used in 10 mol % loading in combination with 10 mol % of TFA as additive, were tested in reaction between cyclohexanone and *p*-nitrobenzaldehyde in DMSO:H_2_O (87:13) as solvent and at room temperature. Catalysts **93a**–**c** showed similar behaviour in terms of conversion, diastereo- and enantioselectivity. Copolymer **93d**, in turn, led to much lower conversion and decrease of stereoselectivity. These results indicated influence on catalytic performance of the functionalization level of the catalytic polymers, since **93d** was much less functionalized than **93a**–**c**.

The catalyst **93b** was also employed in aldol reactions of a wide range of aromatic aldehydes affording the anti-aldol products in high yields and diastereo- and enantioselectivities. Catalyst **94b** provided a series of the aldol adducts with excellent yields and stereoselectivities in a 50:50 mixture of DMF and water as solvent, in shorter reaction times compared to those of catalyst **90b** and without the need for adding acid. The recyclability of polymers **93b** and **94b** was studied in aldol reaction between cyclohexanone and *p*-nitrobenzaldehyde. Copolymer **93b** was recycled seven times affording the aldol product with constant stereoselectivity and with only slight decrease of catalytic activity in every cycle. Catalyst **94b**, on the contrary, was recycled and reused for at least seven runs without any appreciable loss in yield or in stereoselectivity, showing the very positive effect of the *p*-phenylene carboxylate spacer on the chemical stability of the polynorbornene-supported organocatalyst.

**Scheme 33:**
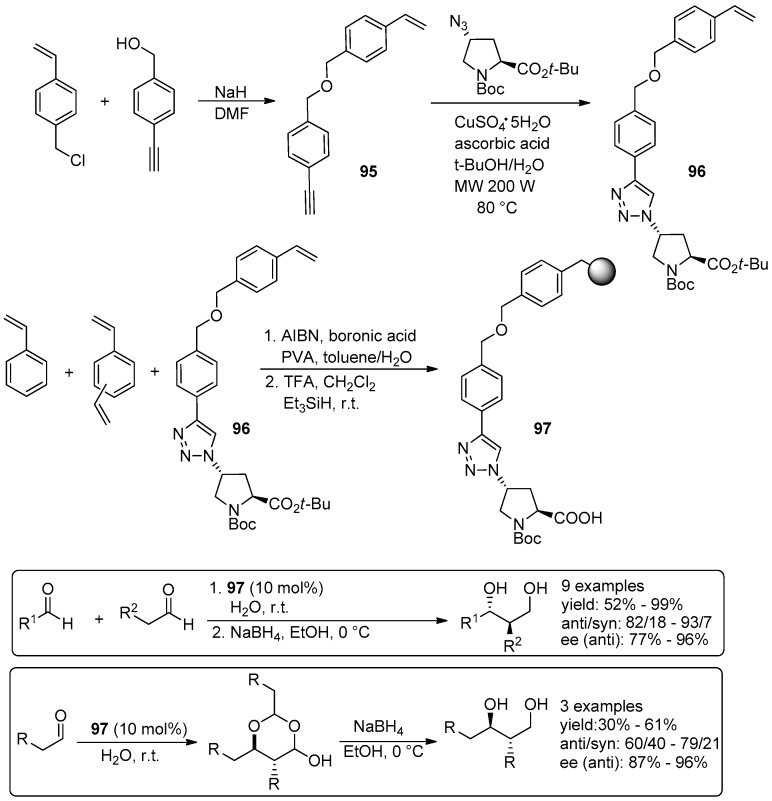
Synthesis of polystyrene-immobilized triazolylproline organocatalyst **97** and its application in the asymmetric cross and self-aldol reactions of aldehydes.

Polystyrene-immobilized triazolylproline organocatalyst **97** was prepared and employed in asymmetric cross- and self-aldol reactions of aldehydes (Scheme 33) [59]. The heterogeneous catalyst **97** was obtained by radical copolymerization of the proline derivative **96** with styrene and DVB and successive deprotection of both the Boc group and the *tert*-butyl ester with TFA. The monomer **96**, bearing a pendant vinyl group, was prepared by click reaction between (2*S*,4*R*)-*N*-Boc-4-azido-l-proline *tert*-butyl ester and bifunctional linker **95** obtained starting from *p*-chloromethylstyrene and 4-ethynylbenzyl alcohol. The asymmetric cross-aldol reaction between a series of aromatic aldehydes and donor aliphatic aldehydes catalyzed by 10 mol % of catalyst **97** in water led to aldol adducts in very good yields and ee values. The same reactions were also carried out in wet DMSO, obtaining an improvement of ee values but a decrease of yield in some cases. In presence of catalyst **97**, the self-aldol reaction of propanal, butanal and 3-phenylpropanal in water as the only solvent gave moderate to good yields and high enantioselectivities. Recyclability of catalyst **97** was studied in the self-aldol reaction of butanal. It was recovered by simple filtration and recycled 10 times showing a moderate decrease of catalytic activity at the end of the recycling test.

**Scheme 34:**
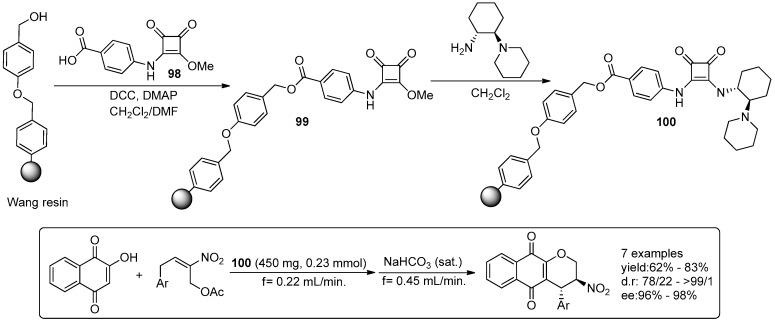
Synthesis of PS-squaramide **100** and its application in the enantioselective flow synthesis of pyranonaphthoquinones.

A new polystyrene-supported squaramide for the enantioselective flow synthesis of pyranonaphthoquinones was reported [60]. In detail, PS-squaramide **100** was prepared following an synthetic strategy that consisted in immobilization of intermediate **98** onto the Wang resin, containing a bis-phenylmethylene ether handle, and in successive nucleophilic displacement with the enantiopure (1*R*,2*R*)-2-(piperidin-1-yl)cyclohexanamine (Scheme 34). After having studied the behavior of resin in batch by the addition of 2-hydroxy-1,4-naphthoquinone to (*E*)-2-nitro-3-phenyl-allyl acetate, the continuous flow experiment was addressed. A new device, in which a packed bed reactor loaded with PS-squaramide **100** was coupled with the T-junction connecting the saturated solution of sodium bicarbonate and the coil reactor, was assembled. A library of enantioenriched pyranonaphthoquinones was prepared by sequential pumping of different solutions containing hydroxynaphthoquinone and a representative set of Morita-Baylis-Hillman acetates. The total residence time of the each process including two sequential reactions—the organocatalyzed Michael addition and the base promoted cyclizations—and in-line work-up was only 30 min. All the pyranonaphthoquinones prepared with this methodology showed excellent stereoselectivities and good yields.

## 4. Metal-Catalysed Non-Asymmetric Reactions

### 4.1. Epoxidation of Alkenes

**Scheme 35:**
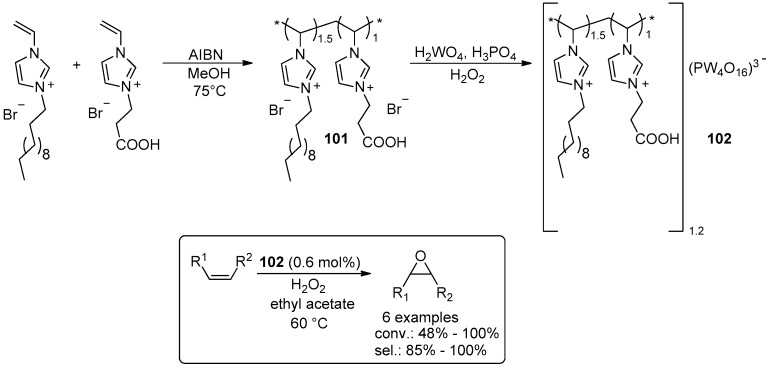
Preparation of POM-paired ionic polymer **102** and its use in the epoxidation of alkenes.

In 2014, Leng et al. reported the first amphiphilic polyoxometalate(POM)-paired ionic polymer-based heterogeneous epoxidation system [61]. The new amphiphilic POM-paired ionic copolymer **102** was prepared by anion-exchange of functionalized-ionic liquid copolymer **101** with the [PO_4_(WO_3_)_4_]^3−^ species (Scheme 35). Copolymer **101** was synthesized by the free radical copolymerization of 3-dodecyl-1-vinylimidazolium bromide and 3-propionic acid-1-vinyl-imidazolium bromide using AIBN as initiator.

The catalytic performance of catalyst **102** was evaluated in the epoxidation of various alkenes with H_2_O_2_ as oxidant and ethyl acetate as solvent. Liquid–liquid–solid three-phase reaction systems were observed and good to excellent conversions (48%–100%) were obtained. The reusability of catalyst **102** was studied in epoxidation of cyclooctene and 1-octene. The catalyst was recovered by filtration and reused for five cycles. A slight gradual loss of activity was observed, while the selectivity was preserved. The results of ICP-AES elemental analysis for the reacted filtrate showed that less than 1.6 wt % of the total W in the catalyst leached into the reaction media. This could have led to the gradual decrease in activity observed in the recycling tests.

**Scheme 36:**
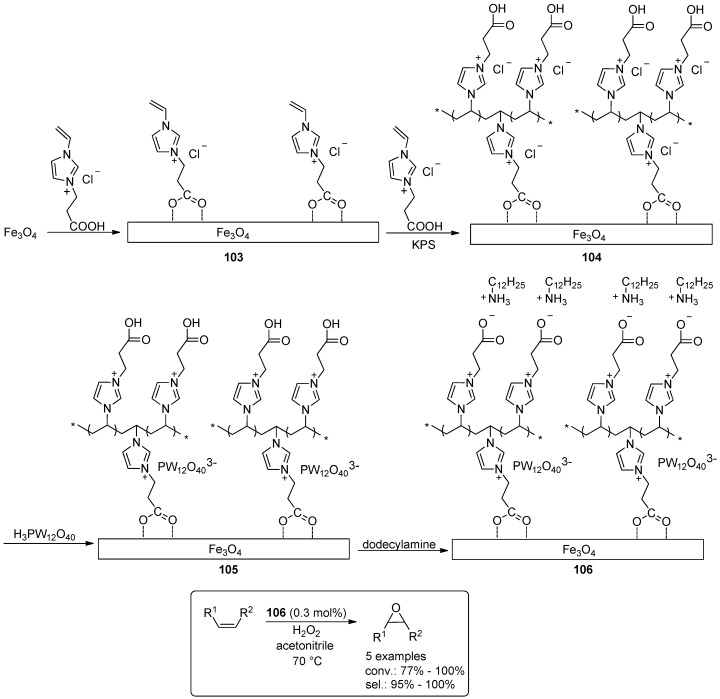
Preparation of catalyst **106** and its application in the epoxidation of alkenes.

In the same year, epoxidation of olefins with H_2_O_2_ was also carried out in presence of the amphiphilic composite **106**, constituted by a magnetic Fe_3_O_4_ core and a dodecylamine-modified polyoxometalate-paired poly-(ionic liquid) shell [62]. The material **106** was prepared in the following way (Scheme 36). First, the 3-propionic acid-1-vinylimidazolium chloride was adsorbed on Fe_3_O_4_ surface via chemisorption interaction between the functional group –COOH and iron ion (see material **103**). Next, the polymerization of the vinyl imidazole ionic liquid in presence of potassium peroxodisulfate (KPS) led to poly-(ionic liquid) coating on Fe_3_O_4_ core **104**.

Afterward, the anion-exchange of poly-(ionic liquid) network with H_3_PW_12_O_40,_ followed by treatment with dodecylamine, provided the material **106**. In presence of **106** as catalyst and acetonitrile as solvent, high conversion values (77%–100%) and selectivities (95%–100%) were obtained. The catalytic reusability was studied in epoxidation of D,L-limonene. After the reaction, the catalyst was easily separated by an external magnet. The recovered catalyst showed a slow decrease in activity (89% conversion) in the third run but the initial catalytic activity (95% conversion) was restored after treatment with dodecylamine. The result of ICP-AES analysis showed that less than 0.9 wt % of W has been leached into the reaction media.

**Scheme 37:**
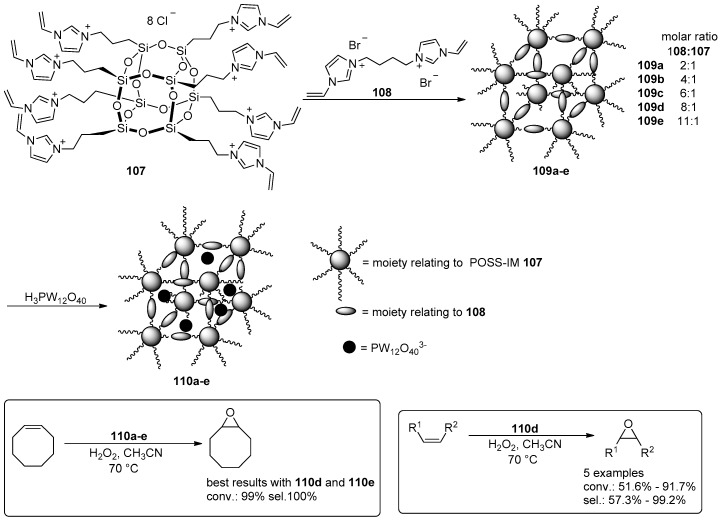
Synthesis of amphiphilic mesostructured polyoxometalate-based ionic hybrids **110a**–**e** and their use as catalysts in the epoxidation of alkenes.

Recent studies have shown that the incorporation of POSS into organic or inorganic polymer could create a micro/mesoporous matrix without using a template [63,64]. In light of this and taking into account that porous framework structure of the solid catalysts would favour the increase of their catalytic performances, Lengh et al. reported the synthesis of POSS-derived mesoporous poly(ionic liquids) **109a**–**e** in order to obtain the amphiphilic mesostructured polyoxometalate-based ionic hybrids **110a**–**e** (Scheme 37) [65]. The introduction of the POSS unit could permit to control the pore structure and wettability of material. The preparation of POSS-derived mesoporous poly(ionic liquids) **109a**–**e** was carried by free radical copolymerization of octa(*N*-vinylimidazole-silsesquioxane)(POSS–IM, **107**) with 1,1-(butane-1,4-diyl)-bis(3-vinylimidazolium) dibromide (**108**), by varying the molar ratio of bis-vinylimidazolium salt **108** to POSS–IM **107**.

This latter was previously synthesized via nucleophilic substitution reaction of chlorine groups of octa(3-chloropropyl silsesquioxane) with vinyl imidazole. Afterwards, the anion-exchange of polymeric frameworks **109a**–**e** with the Keggin-type H_3_PW_12_O_40_ provided mesoporous POM-based ionic hybrids **110a**–**e**. The hybrids **110a**–**e** were applied in H_2_O_2_-based epoxidation of cyclooctene at 70 °C in acetonitrile as solvent. The materials **110d** and **110e**, exhibited the best results (both 99% conversion and 100% selectivity). Furthermore, low conversions were obtained with corresponding POSS-free polyoxometalate-based ionic hybrids and with homogeneous H_3_PW_12_O_40_. The material **110d** was also tested with different substrates obtaining good catalytic activities (51.6%–91.7% conversion) and selectivities (57.3%–99.2%). The catalytic reusability of **110d** was studied in epoxidation of cyclooctene. The catalyst, recovered by filtration, was reused for three run tests without loss of catalytic activity. The result of ICP-AES analysis showed that less than 1.1 wt % W of **110d** has been leached into the reaction media.

**Scheme 38:**
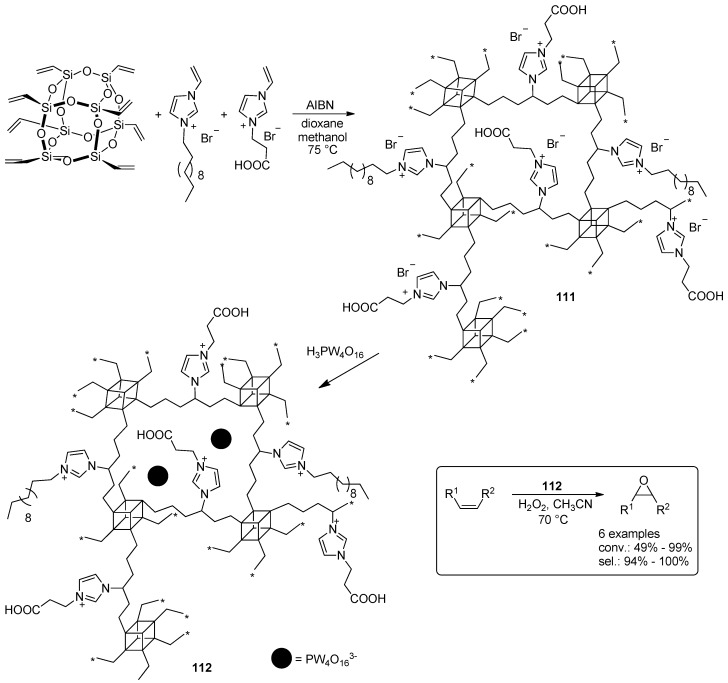
Synthesis of hybrid catalyst **112** and its application in the epoxidation of alkenes.

The study of amphiphilic porous POSS incorporated polyoxometalate-paired polymeric hybrids as catalysts for epoxidation reactions was expanded investigating the synthesis and application of the new hybrid catalyst **112** (Scheme 38) [66]. This material was prepared by radical copolymerization of octavinyl POSS, 3-dodecyl-1-vinylimidazolium bromide and 3-propionic acid-1-vinylimidazolium bromide in 1:3:5 mole ratio, respectively, followed by ion exchange in aqueous solution with the previously prepared H_3_[PO_4_(WO_3_)_4_]. Based on previous studies, this molar ratio permitted to obtain the optimum specific surface (24 cm^2^·g^−1^) and pore volume (0.175 cm^3^·g^−1^). Higher molar ratio of dodecyl imidazole ionic liquid to carboxylic acid functionalized imidazole ionic liquid led to less porous systems, most probably due to the blockage of the channels because of dodecyl groups. Good to excellent activities (49%–99% conversion) and high selectivities (94%–100%) were obtained, applying the catalyst **112** in epoxidation of various alkenes with H_2_O_2_ in acetonitrile as solvent at 70 °C. Finally, the reusability of **112** was evaluated on the epoxidation of cyclooctene. The catalyst was recovered by centrifugation and reused for four runs without observing loss in catalytic activity. Moreover, results of ICP-AES analysis showed that about 3.1 wt % W in the catalyst has been leached into the reaction media in the first run, but in the following runs, the leaching was negligible.

**Scheme 39:**
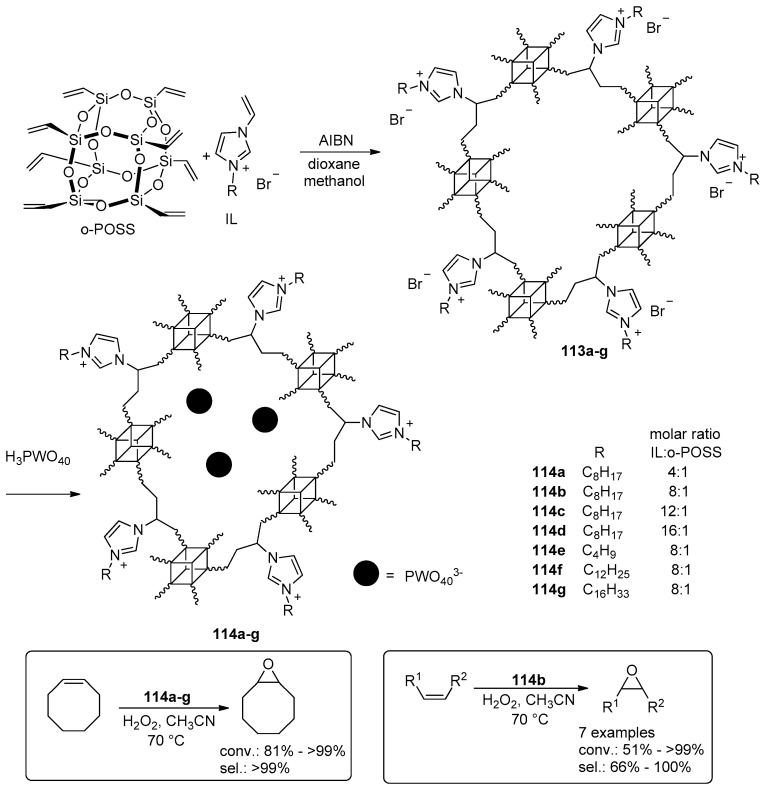
Synthesis of POSS-derived mesostructurated and amphiphilic copolymer-polyoxometalate catalysts **114a**–**g** and their application in the epoxidation of alkenes.

Recently, additional series of POSS-derived mesostructurated and amphiphilic copolymer-polyoxometalate catalysts were successfully synthesized using ionic liquids bearing hydrophobic alkyl chains as the building blocks [67]. Particularly, the catalysts **114a**–**d** were prepared by free radical copolymerization of octavinyl POSS (o-POSS) with ([3-octyl-1-vinylimidazolium]Br) by varying the molar ratio of liquid ionic (IL) to o-POSS and successively carrying out the anion-exchange with H_3_PW_12_O_40_ in aqueous solution (Scheme 39). Catalysts **114e**–**g** were synthesized by copolymerization of o-POSS with imidazole ionic liquids bearing different alkyl chains (C_4_H_9_, C_12_H_25_, C_16_H_33_) keeping the molar ratio IL:o-POSS 8:1 and carrying out the ion-exchange in the same way. The optimum specific surface (25.0 m^2^/g) and pore volume (0.175 cm^3^/g) were obtained with **114b**. The catalytic performances of various catalysts were assessed in the epoxidation of cyclooctene with aqueous H_2_O_2_ as oxidant and acetonitrile as solvent. All catalysts of series showed high conversions (81%–>99%) and selectivity (>99%). Catalyst **114b** was also employed in epoxidation of various alkenes obtaining good to excellent conversions (51%–>99%) and selectivity (66%–100%). The catalytic reusability of **114b** was evaluated in the epoxidation of cyclooctene. The catalyst was recovered by filtration and then reused for four runs without observing loss in catalytic activity.

**Scheme 40:**
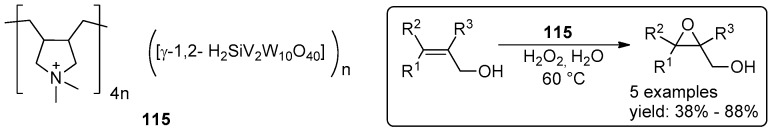
Epoxidation of allylic alcohols catalysed by **115**.

Zhao et al. also reported an effective hybrid catalyst based on polyoxometalate and polymer [68]. This new catalyst, denoted as **115**, was constituted by the linear positively charged polymer, poly(dimethyl diallyl) ammonium, and the polyoxometalate[γ-1,2-H_2_SiV_2_W_10_O_40_]^4−^ (Scheme 40). Catalyst **115** was used in the epoxidation of various allylic alcohols using only one equivalent of hydrogen peroxide in water under mild reaction conditions. *trans*-2-Hexen-1-ol, *cis*-2-penten-1-ol and crotyl alcohol were oxidized to the corresponding epoxides in high yields (79%–88%). Poor reactivity was showed for the oxidation of 2-methyl-2-propen-1-ol (38% yield). Catalyst **115** could be recovered and reused. An emulsion was formed when the catalyst was dispersed in an aqueous reaction system. After the reaction, the organic products were separated from the aqueous phase by extraction with ethyl acetate and the catalyst, remained in the aqueous solution as stable emulsion, could be used for the next run. The reuse of the catalyst for epoxidation of crotyl alcohol was reported; after three consecutive reuses, there was no significant loss of initial catalytic activity. This catalyst was also used in heterogeneous oxidation of sulfides with good results [67].

**Scheme 41:**
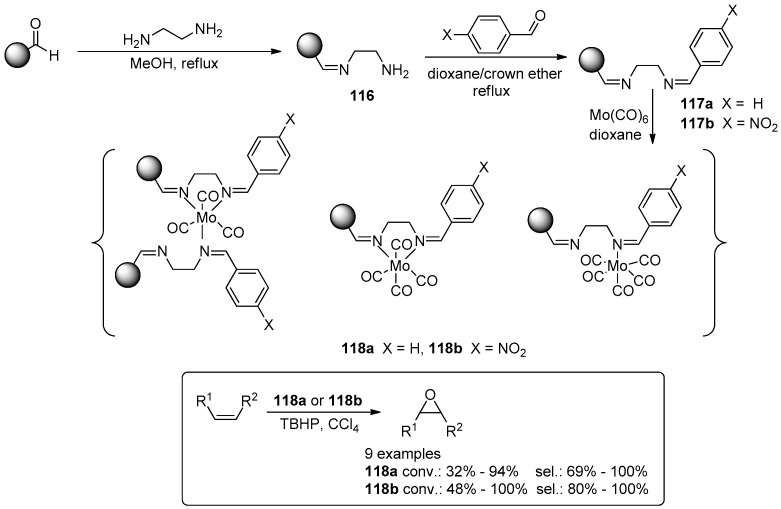
Polymer-supported diimine molybdenum carbonyl complexes **118a**–**b** as pre-catalysts for alkene oxidation.

A wide range of polymer-supported Mo(VI) complexes have been successfully employed as catalysts for alkene epoxidation over the last few decades [69]. In 2014, the synthesis and application of the polymer-supported diimine molybdenum carbonyl complexes pre-catalysts **118a**–**b** (Scheme 41) were described [70]. Molybdenum hexacarbonyl was immobilized onto polystyrene-diimines **117a** and **117b**, that were produced by functionalization of aldehydic polystyrene with ethylene diamine and successive reaction with benzaldehyde or 4-nitrobenzaldehyde. Tetracarbonyl coordination of the supported ligands with the molybdenum carbonyl, the pentacarbonyl and tri-carbonyl coordination of them were assumed. Resins **118a**–**b** were used in the epoxidation of different alkenes under optimized conditions (*tert*-butylhydroperoxide –TBHP– as oxidant and CCl_4_ as solvent), showing to be active and selective. Conversions and selectivities obtained with **118a** were in the 32%–94% and 69%–100% range, respectively, while those obtained with **118b** in the 48%–100% and 80%–100% range, respectively. The reusability of pre-catalysts **118a** and **118b** was investigated in epoxidation of cyclooctene; the pre-catalysts were reused six times without any loss of their initial activities.

**Scheme 42:**
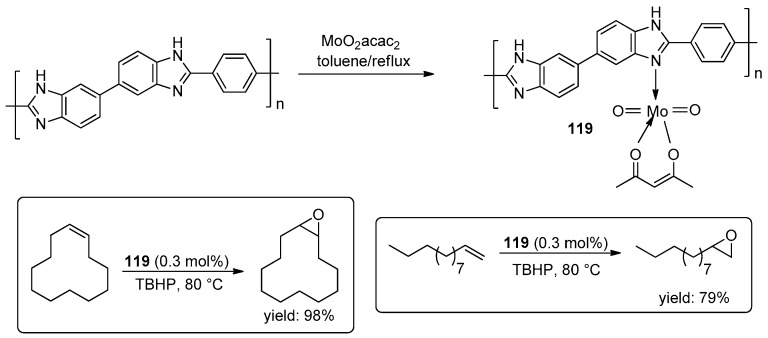
Preparation of polybenzimidazole-supported Mo(VI) complex **119** and its application in the epoxidation of cyclododecene and dodecene.

A new process, which employed polybenzimidazole-supported Mo(VI) complex **119** as catalyst for epoxidation of cyclododecene and dodecene with TBHP as oxidant, was investigated (Scheme 42) [71]. The polymer-supported Mo(VI) complex **119** was prepared through ligand exchange procedure carried out in presence of polybenzimidazole and an excess of molybdenyl acetylacetonate relative to polymer bound ligands under reflux in toluene. The catalytic activity of **119** was studied under different reaction conditions in a batch reactor. The optimum reaction conditions for epoxidation of both cyclododecene and dodecene were found at 80 °C, 0.3 mol % Mo catalyst loading and 2.5:1 feed molar ratio of alkene to TBHP. About 98% yield of 1,2-epoxycyclododecane and about 79% yield of 1,2-epoxydodecane were obtained at 350 min.

The leaching of any catalytically active Mo species from the polymer-supported catalyst **119** was investigated by supernatant studies. The results of supernatant studies of cyclododecene epoxidation revealed that catalytically active Mo species were leached from the polymer matrix, especially at the first three consecutive reactions, causing a decrease of conversion from 96% to 31%. No evidence of Mo leaching was showed for dodecene epoxidation. The authors suggested that the leaching of Mo species from catalyst **119** might be due to the release of Mo species contained in the microgels as a result of mechanical attrition of the polymer catalyst beads. However, they also suggested that this effect would be absent in continuous epoxidation process using a reactive distillation column, as the catalyst would be firmly packed inside a fixed column.

**Scheme 43:**
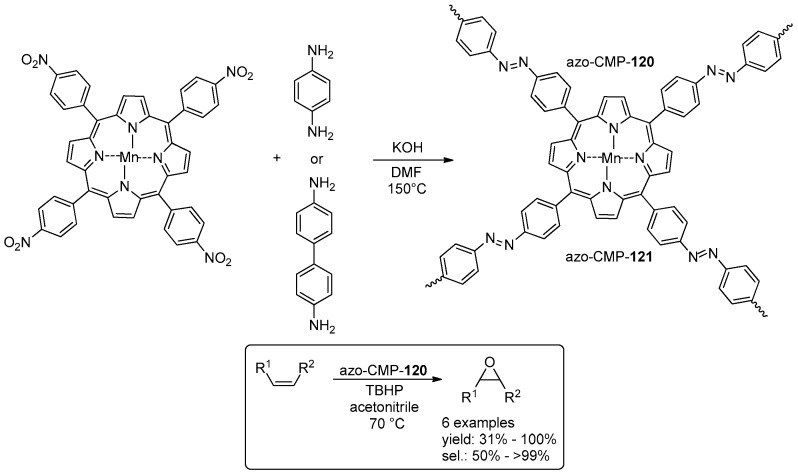
Metalloporphyinic polymers azo-CMP-**120** and azo-CMP-**121** as catalysts for the epoxidation of alkenes.

Zhang et al. described application of two covalent metalloporphyinic polymers (CMPs) based on the azo (–N=N–) linkage, as catalysts for epoxidation of alkenes [72]. These materials, denoted as azo-CMP-**120** and azo-CMP-**121**, were obtained by reaction between manganese *meso*-tetra (4-nitro-phenyl)porphyrin and *p*-phenylenediamine or benzidine respectively, in DMF at 150 °C in the presence of potassium hydroxide under N_2_ atmosphere (Scheme 43).

**Scheme 44:**
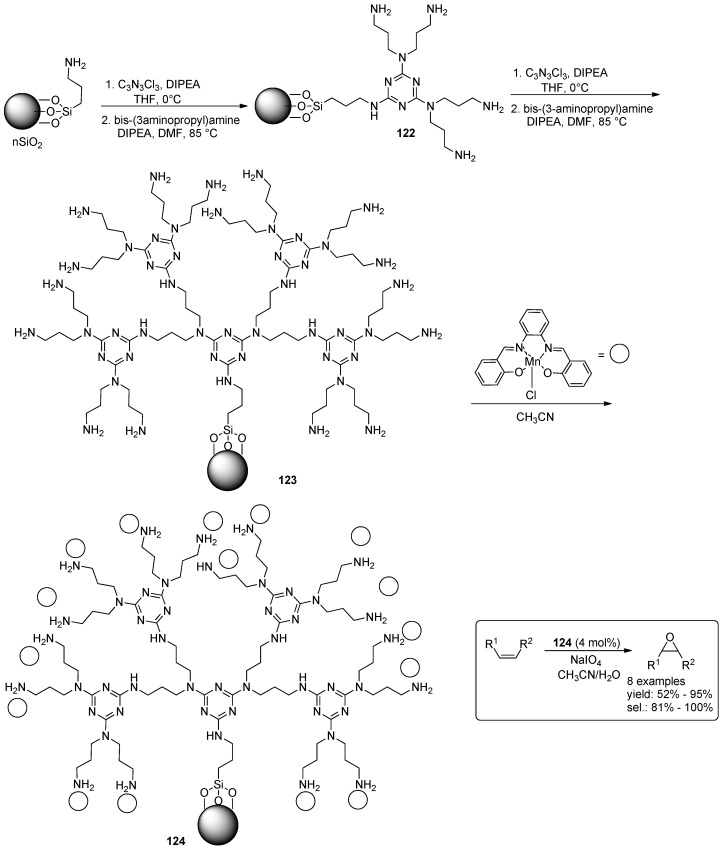
Preparation of Mn(III)salophen supported on nanosilica triazine dendrimer **124** and its catalytic activity in the epoxidation of alkenes.

Activity of catalysts was compared in epoxidation of styrene with TBHP in acetonitrile at 70 °C. The catalyst azo-CMP-**120** displayed a better performance than azo-CMP-**121**. Styrene oxide was obtained with yields of 82% and 69% with azo-CMP-**120** and azo-CMP-**121** respectively. The catalyst azo-CMP-**120** was also employed in epoxidation of other olefins showing high yields with cyclic olefin (79%–100%) and lower yields with linear olefin (35%–58%). Low yield was obtained in reaction with *trans*-stilbene (31%). The catalyst azo-CMP-**120** was recycled five times with a slight decrease in catalytic activity. Recently, Mn(III)salophen supported on nanosilica triazine dendrimer **124** was synthesized and used as new nanocatalyst for epoxidation of different alkenes with NaIO_4_ (Scheme 44) [73].

Catalyst **124** was prepared by coordination reaction of nano-silica-supported triazine dendritic polymer (G2) **123** with Mn(salophen)Cl. Polymer **123** was previously synthesized starting from a propylamine-functionalized nano-silica by using an iterative method that included the nucleophilic substitution reaction between the chloride groups of cyanuric chloride and amino groups of nano-silica followed by reaction with bis(3-aminopropyl)amine [74]. The optimization of epoxidation reaction conditions was studied and several alkenes were oxidized to their corresponding epoxides in good to excellent yield (52%–95%) in CH_3_CN/H_2_O at room temperature with 4 mol % of catalyst **124**. The reusability of **124** was studied in the epoxidation of cyclooctene. The catalyst was separated by simple filtration and reused four runs without significant loss of its initial activity.

### 4.2. C–C Coupling Reactions

**Scheme 45:**
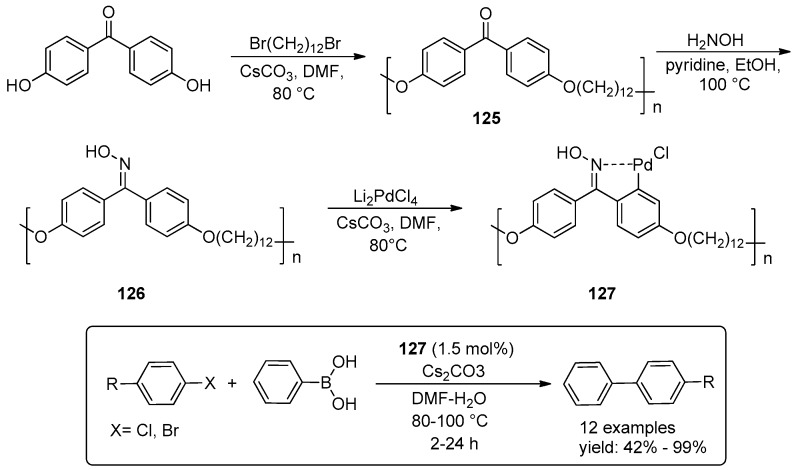
Synthesis of self-supported oximepalladacycle **127** and its application in Suzuki coupling.

In 2014, the self-supported oximepalladacycle **127** was synthesized by using a polymeric ligand based on the polyether ether ketone architecture and employed as pre-catalyst in Suzuki–Miyaura reactions (Scheme 45) [75]. In detail, the self-supported oximepalladacycle **127** was prepared in three synthetic steps: (a) copolymerization of 4,4′-dihydroxybenzophenone with 1,12-dibromododecane to afford **125**; (b) conversion of ketone groups into oximes in order to obtain **126**; and (c) cyclization in the presence of Li_2_PdCl_4_. The Pd loading level was 0.77 mmol/g. Use of **127** as pre-catalyst in the Suzuki–Miyaura reactions between aryl bromides and phenylboronic acid at 80 °C led to biphenyl products with excellent yields (96%–99%) in short reaction times. Higher temperatures and longer times were needed for the reactions with aryl chlorides; only with activated aryl chlorides biphenyl products were obtained in moderate yields (42%–65%). The recyclability of **127** was studied. Catalyst **127** was recovered by filtration, after having cooled the reaction mixture to room temperature and added ether and water to facilitate its precipitation. It was reused up to four times giving excellent yields with low levels of Pd leaching.

**Scheme 46:**
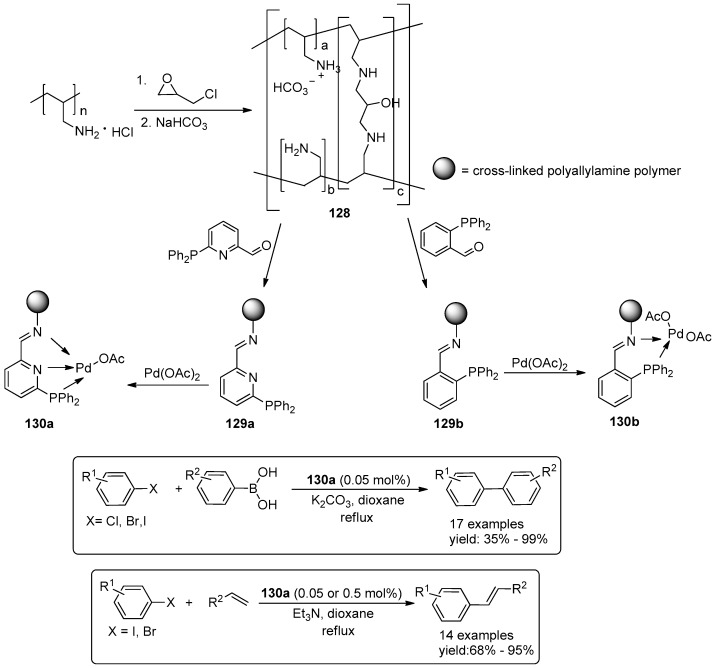
Polymer-supported iminopyridylphosphine palladium(II) complexes **130a**–**b** as catalysts for cross-coupling reactions.

In the same year, cross-linked polyallylamine polymer-supported iminopyridylphosphine palladium(II) complexes were prepared and used as heterogeneous catalysts for Suzuki–Miyaura and Heck cross coupling reactions [76]. These new materials, **130a** and **130b**, were synthesized as depicted in Scheme 46. First, the cross-linked polyallylamine polymer **128** was prepared by copolymerization of polyallyamine hydrochloride salt with epichlorohydrin under basic conditions and successive ion exchange with sodium hydrogen carbonate. Then, the amino-groups of **128** were functionalized by 6-(diphenylphosphino) picolinaldehyde or 2-(diphenylphosphino)benzaldehyde to form cross-linked polyallylamine polymer-supported phosphine–Schiff bases **129a** or **129b** respectively. Finally, the complexation of **129a** and **129b** with Pd led to materials **130a** and **130b** (Pd loading **130b**: 0.57 mmol/g; Pd loading **130a**: and 0.5 mmol/g). The catalytic activity of **130a** and **130b** was compared in the coupling of bromobenzene with phenylboronic acid under optimized conditions (dioxane, K_2_CO_3_, 0.05 mol % of catalyst, reflux). Catalyst **130a** was found to be more efficient than catalyst **130b.** Catalyst **130a** was also employed in Suzuki reaction between different aryl halides and substituted phenylboronic acids. High yields were obtained with aryl iodides and bromides (90%–99%). Low to high yields with aryl chlorides (35%–95%) were obtained. The reaction between 4-amino-1-chlorobenzene and phenylboronic acid only gave 35% of desired product, while the reaction between 4-chloronitrobenzene and 4-methoxyphenylboronic gave better result (yield 95%). The use of catalyst **130a** in Heck reaction of aryl halides with terminal olefins also gave good yields (68%–95%). Recycling experiments were carried out for the cross-coupling of bromobenzene and phenylboronic acid. The catalyst was recovered by filtration and reused at least five times without significant loss of activity.

**Scheme 47:**
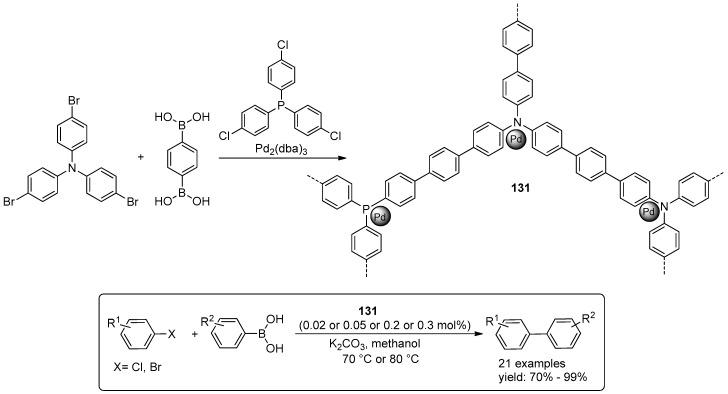
Synthesis of Pd heterogeneous catalyst **131** and its application in Suzuki–Miyaura reaction.

Recently, triphenylphosphine ligand and palladium nanoparticles were immobilized in situ in a polymer synthesized by palladium catalysed Suzuki–Miyaura reaction between tris(4-bromo-phenyl)amine and benzene-1,4-diboronic acid, to which tris(4-chlorophenyl) phosphine participated [77]. The optimization of this procedure by using Pd_2_(dba)_3_ led to Pd heterogeneous catalyst **131** (Scheme 47). The amount of Pd in the catalyst, determined by ICP-AES, was 1.81 wt %. Application of catalyst **131** in Suzuki–Miyaura reaction of aryl chlorides and bromides with aryl boronic acids allowed to obtain the corresponding biphenyl compounds in good to excellent yields (70%–99%). The reusability of the catalyst was tested in the cross coupling of 4-chlorotoluene with phenylboronic acid. It was reused five times; a slight deactivation of catalyst was observed.

**Scheme 48:**
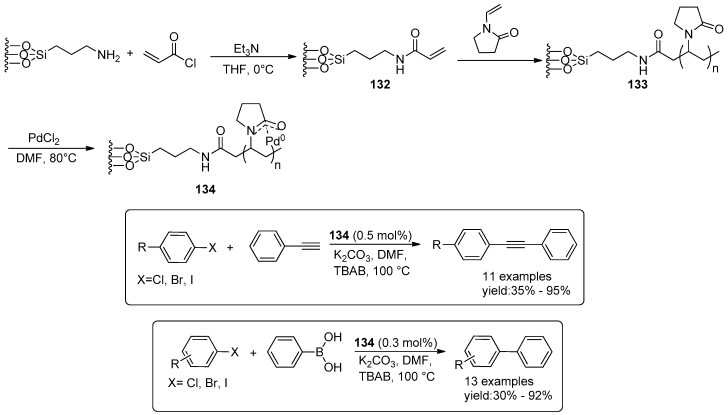
Preparation of heterogeneous catalyst **134** and its use in cross-coupling reactions.

The application of Pd nanoparticles supported on poly(*N*-vinylpyrrolidone)-grafted silica as heterogeneous catalysts for Sonogashira and Suzuki coupling reactions was also reported [78]. In detail, catalyst **134** was obtained by treatment of poly(*N*-vinylpyrrolidone)-grafted silica **133** with PdCl_2_ in DMF (Scheme 48). Support **133** was afforded by reaction between acryloyl chloride and aminopropyl silica and subsequent copolymerization of the thus obtained material **132** with 1-vinyl-2-pyrrolidone in the presence of benzoyl peroxide as initiator.

Catalyst **134** contained an average of 0.25 mmol·g^−1^ of Pd. Under optimized conditions (0.5 mol % of catalyst, 2 eq. K_2_CO_3_, DMF, 100 °C and tetrabutylammonium bromide (TBAB) only for reactions with aryl chlorides), Sonogashira-Hagihara cross-coupling reaction between phenylacetylene with different aryl halides was carried out. High yields were obtained unlike the less active 4-chloroanisole, which led to coupling product in 35% of yield. Catalyst **134** was recovered by filtration and recycled successfully for seven runs. A total Pd leaching of about 3% from the supported system after the third and sixth run was observed. Use of catalyst **134** in Suzuki coupling reaction between phenylboronic acid and different aryl halides, in the presence of K_2_CO_3_ as base and DMF as solvent, provided biaryls with good yields. *Ortho-* and *meta-*substituted aryl halides gave the corresponding products after 24 with only 30% and 40% of yield. Similar to the Sonogashira reaction, aryl chlorides gave the coupling products in the presence of TBAB. The recycling experiments for the reaction of phenylboronic acid with iodobenzene were carried out; the catalyst was used six times with no considerable leaching.

**Scheme 49:**
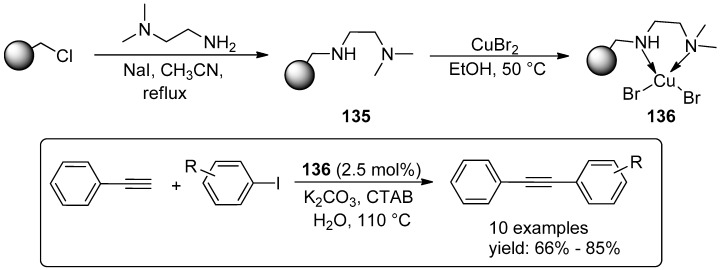
Polystyrene-supported Cu(II) complex **136** as catalyst for Sonogashira reaction.

Kodicherla et al. reported the first Sonogashira coupling reaction catalysed by polystyrene-supported Cu(II) complex [79]. The polystyrene-supported Cu(II) *N,N*-dimethyl-ethylenediamine complex **136** was prepared by treatment of polymer-bound *N,N*-dimethylethylenediamine **135** with an ethanolic solution of CuBr_2_ at 50 °C (Scheme 49). Polymeric ligand **135** was synthesized by reaction between chloromethylated polystyrene and *N,N*-dimethylethylenediamine in refluxing acetonitrile. The amount of copper incorporated into the polymer, determined by atomic absorption spectroscopy, was 0.98 mmol/g. Application of catalyst **136** in Sonogashira cross-coupling reaction between phenylacetylene and aryl iodides containing electron withdrawing or donating substituents gave the corresponding biarylacetylenes in good yields (66%–85%). The reusability of the catalyst **136** was studied in reaction between iodobenzene and phenylacetylene. Not much decrease in the activity of catalyst was observed even after four cycles. During the course of Sonogashira coupling reactions, 0.4% of copper was lost into solution after the first run. After four recycles loss of 7% was observed. This catalyst was also applied in *N*-arylation of indoles [79].

**Scheme 50:**
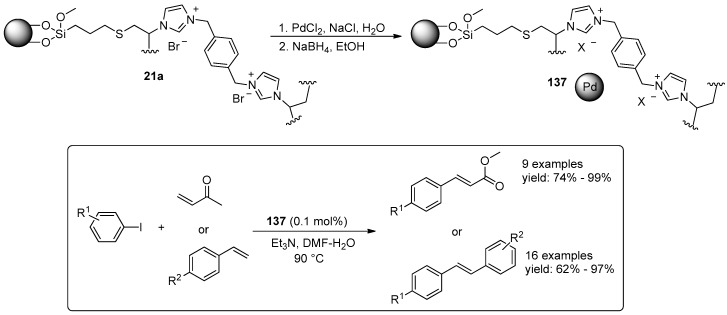
Palladium on highly cross-linked imidazolium-based material **137** as catalyst for Heck reaction.

In 2011, a new concept of heterogeneous materials based on highly cross-linked imidazolium salts anchored on silica gel as supports for palladium nanoparticles in high loadings was developed [80]. This topic was extended with the preparation of the material **137** as catalyst for the Heck reaction (Scheme 50) [81]. Material **137** was prepared by treatment of highly cross-linked imidazolium-based support **21a** with an aqueous solution of Na_2_PdCl_4_, followed by reduction reaction with NaBH_4_ in ethanol. Material **137** was employed in 0.1 mol % in Heck reaction between styrenes or methyl acrylate and various aryl iodides in DMF/H_2_O 4:1 in the presence of TEA at 90 °C, showing good results. Under these conditions, catalyst **137** released Pd species in solution and the leached Pd species catalysed the reaction. Adding a six-fold amount of the imidazolium-based support **21a**, they were very efficiently scavenged. The new supported Pd species (0.5–0.6 wt %) were not catalytically active. The non-catalytic activity of low loaded Pd material was explained assuming that such Pd species were strongly bound into the inner surface of the multilayered structure of support **21a**. The use of the highly cross-linked imidazolium-based materials as palladium scavengers was also extended [82].

**Scheme 51:**
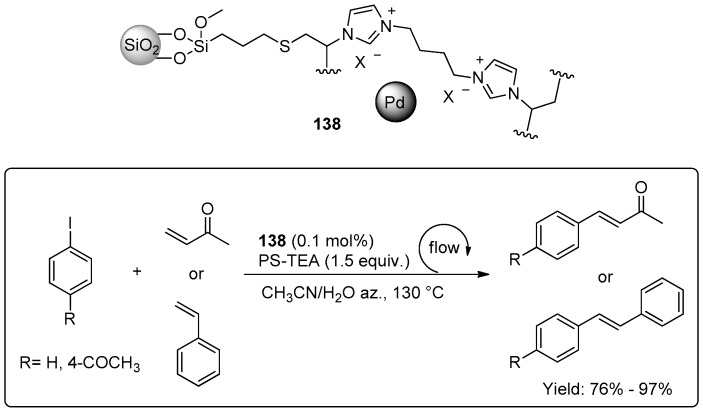
Palladium on highly cross-linked imidazolium-based material **138** as catalyst for Heck reaction in flow.

A novel and highly sustainable protocol for flow Heck reaction in presence of the catalyst **138**, constituted by palladium (10 wt %) supported on cross-linked imidazolium network, was reported (Scheme 51) [83]. Flow reactors and protocols were defined in order to optimize the recovery and reuse of the catalyst, minimize waste and in particular, the amount of organic solvent needed to isolate the final products. The flow protocol was defined by charging a mixture of the catalyst **138** (0.1 mol %) and the supported base, diethylaminomethyl-polystirene (PS-TEA) (1.5 equiv.), in a stainless steel HPLC column, while the reactants solution was charged in another glass column, acting as the reservoir. After setting the temperature at 130 °C, the reaction mixture was cyclically pumped through the columns at a 2.0 mL/min flow rate for the time required to obtain quantitative conversion. Reusability of catalyst **138** was investigated in Heck reaction to methyl cinnamate. The reuse of the catalyst was repeated for four consecutive runs, achieving a TON of about 4000 and a TOF of about 0.08 s^−1^. No apparent activity loss was observed and a Pd content of about 4 ppm was found in the products obtained after each cycle of the flow protocol. Furthermore, the CH_3_CN aqueous azeotrope used in the reaction was distilled off and recovered with a high purity (GLC analysis), totally comparable to the starting azeotropic mixture, thus being reusable without affecting the reaction yield. The flow procedure at 130 °C was extended to the reactions between two aryl iodide and styrene or methyl acrylate, obtaining good yields (76%–97%) and Pd content into the products <5 ppm. The combination of the flow technique and sustainable reaction conditions led to a substantial reduction of the waste production, as proved by the low E-factor value of 12.

**Scheme 52:**
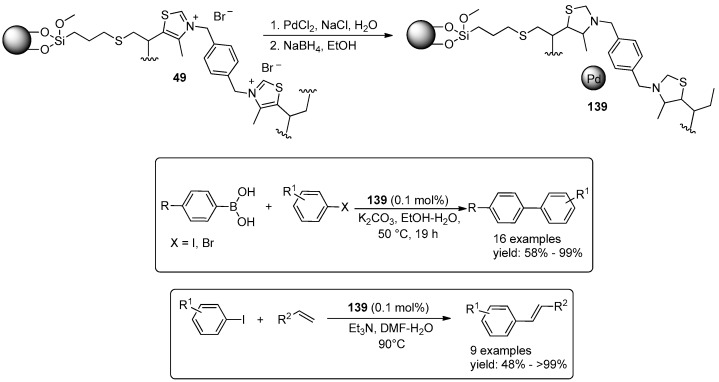
Palladium on highly loaded thiazolidine-functionalized silica **139** as catalyst for Suzuki and Heck reactions.

Taking advantage of application of highly cross-linked imidazolium-based materials as supports for palladium nanoparticles, another approach for the palladium immobilization, based on the highly loaded thiazolidine-functionalized silica support **49**, was described (Scheme 21) [84]. The thiazolidine-based support **49** was treated with an aqueous solution of Na_2_PdCl_4_ and successively with sodium borohydride, to afford the catalyst **139** (Scheme 52). Catalyst **139** in 0.1 mol % loading was used in the Suzuki–Miyaura reaction between phenylboronic acid or 4-formylphenylboronic acid and a set of aryl bromides (or iodides) in ethanol/water at 50 °C in the presence of K_2_CO_3_ as base. Biphenyl compounds were obtained in high yields (58%–99%). The catalyst was also used in the Heck reaction between aryl iodides and methyl acrylate or styrene. Good to excellent yields were obtained, except in the reaction between 2-iodothiophene and methyl acrylate. The recyclability was studied in Suzuki reaction. The catalyst was used three times without loss of catalytic activity.

**Scheme 53:**
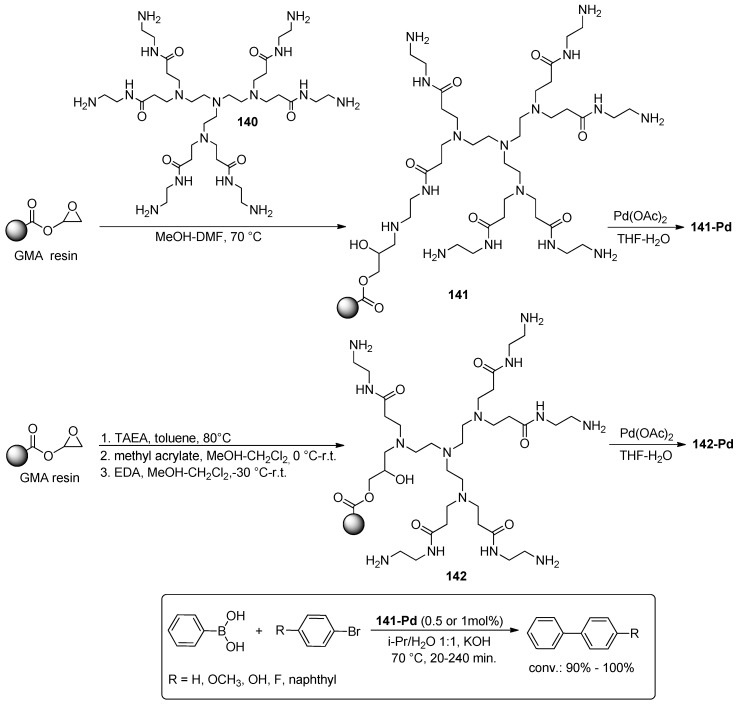
Palladium on supported PAMAM, **141-Pd** and **142-Pd**, for Suzuki coupling.

New polymer supports for palladium catalysts were prepared by immobilization of poly(amidoamine) PAMAM type hyperbranched systems on an epoxy-functionalized polymer(GMA resin) derived from terpolymers of glycidyl methacrylate, styrene, and divinylbenzene [85]. The supported PAMAM **141** and **142** were synthesized considering two methods (Scheme 53). The first included the immobilization of the zeroth order PAMAM dendrimer **140** [86] on the GMA resin. The second relied on multi-stage chemical modification of the GMA resin in turn with tris(2-aminoethyl)-amine (TAEA), methyl acrylate and ethylenediamine (EDA). Palladium(II) ions were immobilized onto supported PAMAM **141** and **142** thus obtaining the catalysts **141-Pd** and **142-Pd** with Pd(II) loading of 16.3 and 8.1% Pd(II) ions, respectively. Polymer-supported Pd(II) complexes **141-Pd** and **142-Pd** were tested as catalysts in the Suzuki–Miyaura reaction. Kinetic plots obtained for the reaction of bromobenzene with phenylboronic acid performed in the presence of **141-Pd** and **142-Pd**, in i-PrOH–H_2_O mixture, at 70 °C, using KOH as base and 0.5 mol % of catalyst, showed that **141-Pd** was more active than **142-Pd**. Therefore, **141-Pd** was used for the further catalytic studies with a series aryl bromides and phenylboronic acid obtaining high conversions (90%–100%). An incomplete conversion (about 90%) was noted only in the case of *p*-bromophenol. The recyclability of catalyst **141-Pd** was studied in the reaction of *p*-bromoanisole with phenylboronic acid. The catalyst beads were reused nine times without regeneration. Despite, the loading of palladium diminished in about 9% after 5th run and about 12.5% after 10th run, the efficiency of **141-Pd** was not influenced.

**Scheme 54:**
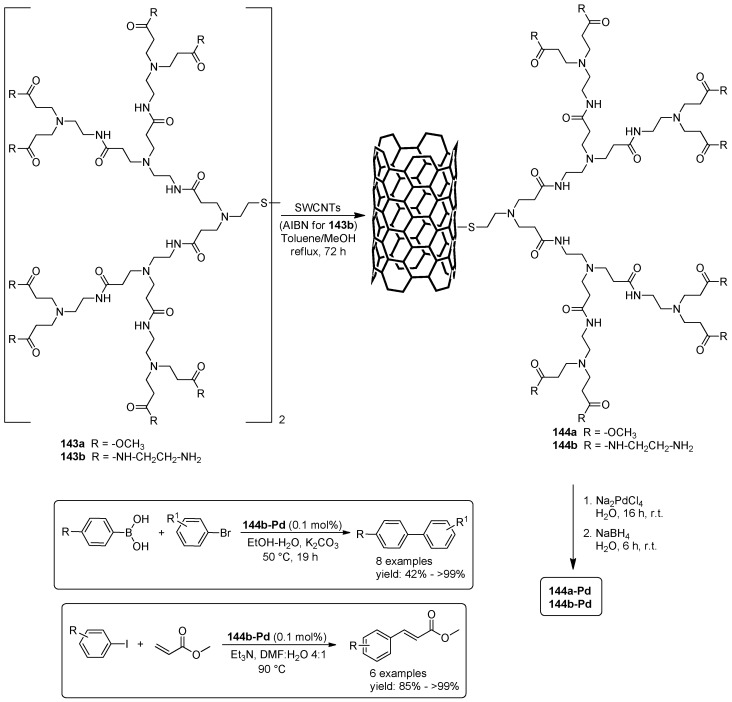
Synthesis of SWCNT-PAMAM-Pd hybrids **144a**–**b-Pd** and their use in cross coupling reactions.

Recently, polyamidoamine dendrimers (PAMAM) were also immobilized onto single-walled carbon nanotubes (SWCNTs) to obtain suitable supports for palladium nanoparticles [87]. In detail, hybrid supports SWCNT–PAMAM **144a** and **144b** were prepared by direct reaction between pristine SWCNTs and cystamin-based PAMAM dendrimers 2.5 G **143a** and 3.0 G **143b**, respectively (Scheme 54). Then, heterogeneous catalysts **144a-Pd** and **144b-Pd** were obtained by treatment of **144a** and **144b** with [PdCl_4_]^2−^ and successive reduction with sodium borohydride, respectively. By this strategy, materials **144a** and **144b** were uniformly decorated with small palladium nanoparticles with mean diameters of 3.2 and 1.6 nm, respectively, in 10 wt % loading of Pd. Both catalysts were tested in Suzuki–Miyaura and Mizoroki–Heck reactions and catalyst **144b-Pd** showed the best performances. The authors proposed a “release and catch” mechanism [88] for both reactions. During the Suzuki reaction, the re-deposition of soluble Pd-species was highly effective. During the Heck coupling instead, part of the soluble Pd species was stabilized by both the base and the solvent and subsequently leached away. The reusability of nanocatalyst **144b-Pd** was also studied. It was shown to be recyclable at least four times in the Suzuki reaction and six times in the Heck coupling with quantitative yields.

**Scheme 55:**
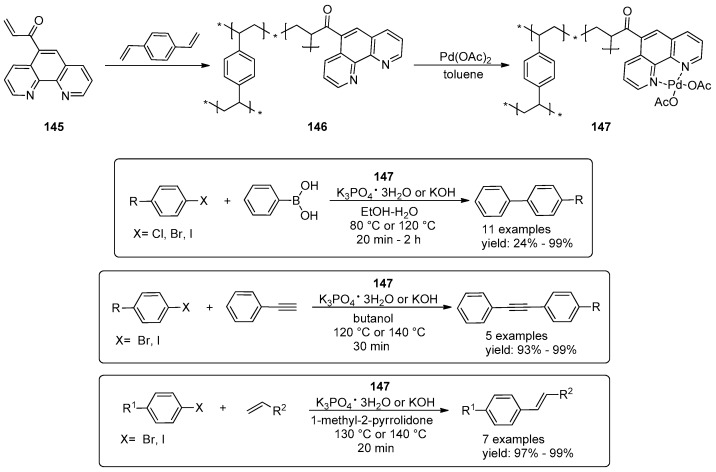
Preparation of polymer-supported Pd-catalyst **147** and its application in cross coupling reactions.

A novel strategy for the preparation of highly efficient Pd-based heterogeneous catalyst, using a porous organic polymer containing phenanthroline ligands, was reported [89]. Particularly, catalyst **147** was obtained by metalation with Pd(OAc)_2_ of polymer organic **146** synthesized through the copolymerization of divinylbenzene and vinyl-functionalized phenanthroline monomer **145** under solvothermal conditions (Scheme 55). The activity of catalyst **147** was tested in Suzuki, Heck and Sonogashira reactions. The Suzuki coupling between phenylboronic acid and aryl bromides or aryl iodides at 80 °C gave biphenyl products with excellent yields (94%–99%). In presence of less reactive aryl chlorides, relatively low yields (24%–34%) were obtained even when the reactions were carried out at a higher temperature (120 °C), over a longer reaction time and with a higher catalyst loading (0.05 g instead of 0.01 g for 1 mmol of halide). The Sonogashira coupling between benzyne and aryl bromides or aryl iodides at 120 °C gave the corresponding biarylacetylenes in high yields (93%–99%) without CuI co-catalyst. In the case of Heck coupling, catalyst **147** again exhibited very high activity and selectivity, converting aryl iodides and bromides with high yields (97%–99%). In all reactions, material **147** was more active than the corresponding Pd-based homogeneous catalyst used under the same conditions. Furthermore, the catalyst was found to be recyclable. It was used for five recycling trials without significant loss of the catalytic activity and leaching of Pd. According authors, this result might be reasonably attributed to the strong coordination between the Pd species and the phenanthroline moieties.

**Scheme 56:**
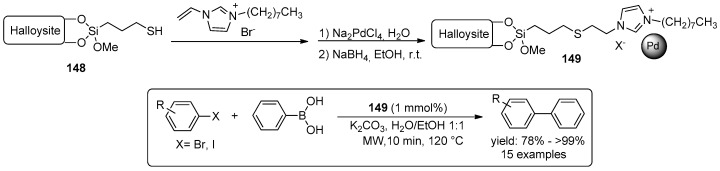
Preparation of material **149** and its application in Suzuki reaction.

Halloysite, a double-layered aluminosilicate mineral that has a predominantly hollow tubular structure, has been also used as support for ionic liquid (IL) phase-based materials. External surface functionalization of halloysite nanotubes (HNTs) with ILs was achieved through grafting of 3-mercaptopropyl trimethoxysilane by microwave irradiation, followed by anchorage of 3-octyl-1-vinylimidazolium bromide via thiol-ene reaction (Scheme 56) [90,91]. MW irradiation allowed obtaining high loading onto the HNTs surface compared to those obtained through conventional synthesis. Treatment with Na_2_PdCl_4_ in water followed by reduction with NaBH_4_ gave the Pd-based catalyst **149**. This latter was used in Suzuki reactions between phenylboronic acid and several aryl halides in ethanol/water with a catalytic loading of 1.0 or 0.1 mol %. Reactions were carried out under MW irradiation at 120 °C for 10 min to give the corresponding biphenyls in good to excellent yields. Up to five cycles were performed using catalyst **149** at 1.0 mol % loading.

**Scheme 57:**
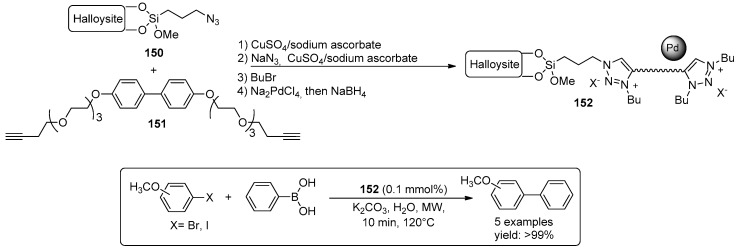
Preparation of material **152** and its application in Suzuki reaction.

In addition, the Pd-based halloysite-bis-triazolium salt **152** was prepared. In this case, the starting material was the azido-functionalized HNTs **150**. The HNTs modified with the bis-triazolium salt were obtained using the bis-alkyne **151** and a reactions sequence based on two click reactions, first with the azido-HNT **150**, then with NaN_3_, followed by quaternization with 1-bromobutane (Scheme 57) [92]. Palladium was immobilized from Na_2_PdCl_4_ solution followed by reduction. Catalyst **152** was used (0.1 mol %) in the Suzuki–Miyaura reaction between phenylboronic acid and a set of aryl halides in water at 120 °C in the presence of K_2_CO_3_ as base. All the reactions were run for 10 min under MW irradiation. High conversions were obtained with anisole derivatives, lower yields with other substrates but an increasing in their conversion was achieved carrying out a reaction in mixture water/ethanol (1:1). Recycling of catalyst was investigated by the reaction between phenylboronic acid and 3-bromoanisole. Catalyst was recovered by centrifugation and reused in the same reaction in five cycles. Biphenyl-3-anisole was afforded in 99%–90% for five cycles.

**Scheme 58:**
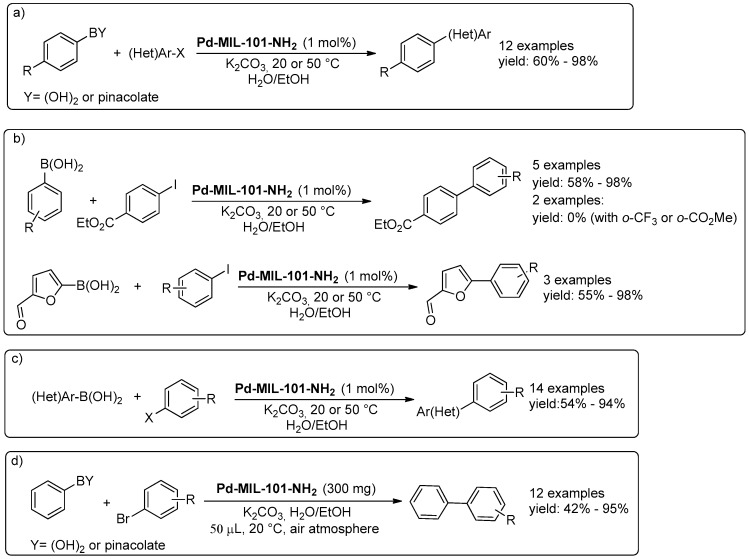
Synthesis of highly-functionalized biaryls via Suzuki–Miyaura cross-coupling catalyzed by **Pd-MIL-101-NH_2_**.

In 2015, the synthesis of highly-functionalized biaryls via Suzuki–Miyaura cross-coupling catalyzed by Pd nanoparticles supported on the amino-functionalized mesoporous metal–organic framework MIL-101-NH_2_ (**Pd-MIL-101-NH_2_**, see Scheme 58) was proposed [93]. This catalytic system with a palladium loading of 8 wt % was known to have the optimal amount of palladium impregnated in MIL-101-NH_2_ [94,95] for this type of applications [96]. In presence of 1 mol % of **Pd-MIL-101-NH_2_**, the coupling of boronic acids or pinacolate esters with heteroaryl iodides and bromides was investigated. Heteroatom-functionalized biaryls were obtained in excellent yields at mild temperatures (20 or 50 °C) and in very short reaction times (10 min–4 h) (Scheme 58a). Influence of steric effect given by substitution in phenylboronic acids or aryl halides was also evaluated. For this reason, a series of reactions was carried (Scheme 58b). High yields were obtained with all substituted boronic acids, except that with boronic acids containing electron-withdrawing and bulky *ortho* substituents with which the desired products were not obtained. The presence of bulky and electro-withdrawing *ortho* functionality in aryl halide determined only a slight decrease of yield. Superior results were obtained when the heterocyclic moiety belonged to the boron transmetallation partners (Scheme 58c); a better tolerance towards multiple coordinating heteroatoms was noticed. The Pd leaching was investigated observing its dependence on the nature of the substrates and the reaction condition. In absence of heteroatoms in substrates, the Pd content in solution after filtration of the catalyst was in most cases <0.1 ppm. However, when one N atom was present in the aryl halides, leached Pd amount was very small (2.8–5.0 ppm) and proved to be dependent on the reaction time and temperature. At elevated temperatures and under MW irradiation, even higher levels of Pd could be detected (12–14 ppm). After having studied **Pd-MIL-101-NH_2_** under batch conditions, its behavior in continuous-flow conditions was investigated. For the first time, metallic nanoparticles supported on metal–organic framework were employed in a packed-bed micro-flow reactor for catalytic applications. Twelve biaryls were synthesized without replacing the catalyst, demonstrating the potential of the catalyst for large-scale applications (Scheme 58d). The catalyst showed a good stability under a continuous flow regime with very slight leaching of metallic species.

### 4.3. Miscellaneous Examples

**Scheme 59:**
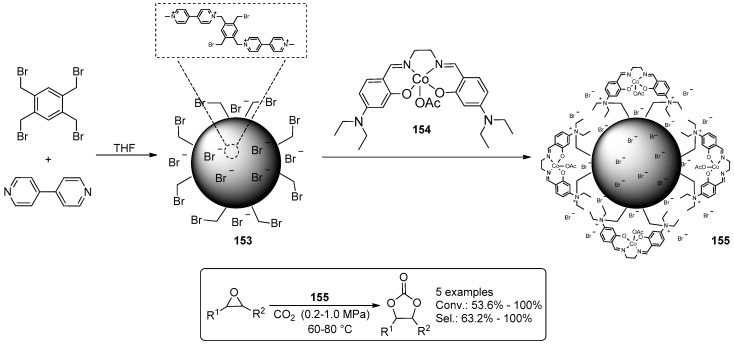
Synthesis of TBB-Bpy@Salen-Co **155** and its application in the cycloaddition of CO_2_ to epoxides.

In order to increase the catalytic activity toward the synthesis of cyclic carbonate, by cycloaddition reaction of CO_2_ to epoxides, bifunctional catalysis based on both Lewis acids and quaternary ammonium salts can be used. Ionic polymer microsphere (see also Scheme 10) bearing Co(III)-Salen moiety were used for this scope [97]. Particularly, the new bifunctional solid catalyst TBB-Bpy@Salen-Co **155** was synthesized by grafting a Salen-Co III Schiff base **154** onto cross-linked TBB-Bpy ionic polymer **153**, prepared by reaction between TBB (1,2,4,5-tetrakis(bromomethyl)-benzene,) and Bpy (4,4′-bipyridine) in a one-pot solvothermal method (Scheme 59).

The catalyst was applied in the cycloaddition reaction of CO_2_ to various epoxides at 60 °C–80 °C, even at low CO_2_ pressure (0.2 MPa) under solvent-free conditions and without any additives. The corresponding cyclic carbonates were obtained with high conversions and excellent selectivities. The TON was approximately 300–500 moles of product per mole of catalyst based on Co. A possible synergistic mechanism that involved the active sites of catalyst functionalized with Co atoms and Br was proposed. The catalyst recyclability was investigated by cycloaddiction of propylene oxide. The solid catalyst was recovered by filtration and reused for five cycles without any significant loss of activity or selectivity. ICP-AES elemental analysis revealed 4.62 wt % Co in the catalyst recovered after five runs, which was very similar to that found for the fresh catalyst (4.71 wt % Co), thus indicating the heterogeneous nature of the catalyst.

**Scheme 60:**
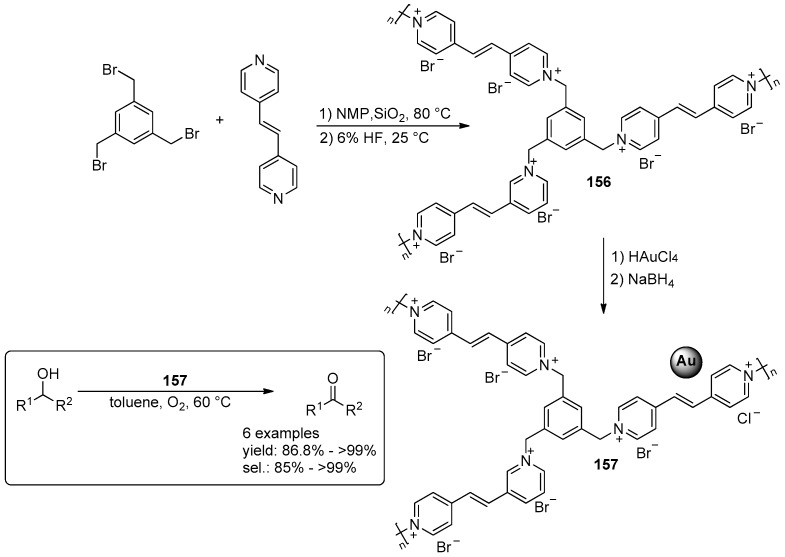
Synthesis of gold nanoparticles supported on nanoporous ionic organic network **157** and its use as catalysts for alcohol oxidation.

Similar ionic polymers were also used to stabilize AuNPs for oxidation reactions. Zhang et al. reported the application of gold nanoparticles supported on a nanoporous ionic organic network (PION) as catalysts for alcohol oxidation (Scheme 60) [98]. For this reason, the PION **156**, having a high ionic density (three cation–anion pairs per unit), was synthesized by nucleophilic substitution between commercial 1,3,5-tris(bromomethyl)benzene and 1,2-bis(4-pyridyl)ethylene. Gold nanoparticles (Au NPs) were supported on PION **156** by ion exchange reaction with AuCl_4_−ions and successive chemical reduction with freshly prepared aqueous solution of NaBH_4_. The new hybrid material thus obtained, denoted as **157**, presented quite small Au NPs (mean size: 2.2 nm) and homogeneously dispersed on the polymer support. In presence of catalyst **157** and in toluene as solvent, the aerobic oxidation of a saturated alcohols was carried out affording the corresponding ketones with high yields and selectivities.

The recyclabily of catalyst **157** was investigated by cyclohexanol oxidation. It was recovered by centrifugation and reused over five runs maintaining its catalytic activity. Furthermore, the liquid phase of the reaction mixture was also collected by hot filtration after the first run and analyzed by inductively coupled plasma mass spectrometry. A very low amount of dissolved gold (0.1% of the total gold) was detected in the solution at the end of the reaction.

**Scheme 61:**
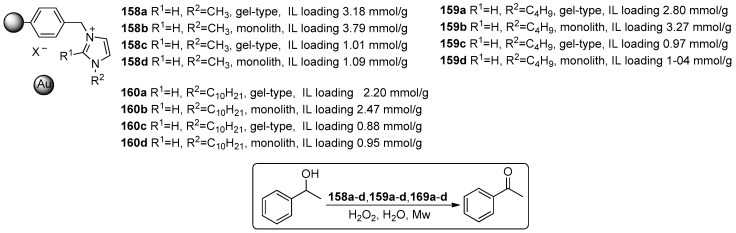
AuNPs-SILLPs **158a**–**d**, **159a**–**d**, **160a**–**d** as catalysts for microwave-assisted selective oxidation of 1-phenyl-ethanol in water.

Restrepo et al. investigated the efficiency of twelve different composites (**158a**–**d**, **159a**–**d**, **160a**–**d**), constituted by gold nanoparticles immobilized onto imidazolium-based supported ionic liquid like phases (AuNPs-SILLPs), as catalysts for microwave-assisted selective oxidation of 1-phenyl-ethanol in water with H_2_O_2_ as oxidant (Scheme 61) [99]. SILLPs with different variables [100,101], including the functionalization degree, resin morphology (gel-type vs. macroporous resins) and the alkyl substitution of the imidazolium moiety (–Me, –But, or –Dec), were evaluated. The effect of these different supports on the mean reaction rate was studied by a Design of Experiments (DoE) based on the Taguchi methods [102], carrying out the 1-phenylethanol oxidation reaction at two different temperatures (100 and 150 °C) and several reaction times (15, 30, 60 and 120 min). The DoE analysis suggested that the increase of the chain length on the imidazolium, following the order –Me, –But and –Dec, led to an enhancement of the reaction rate. This trend also matched the one observed for AuNPs size distribution. Increasing the length of alkyl chain, a bigger AuNPs size distribution was observed. The larger reaction rates were found for AuNPs-SILLPs based on macroporous resins. The effect of the ionic liquid loading was the least pronounced. The DoE analysis also indicated that in many cases there was an enhancement of the catalytic efficiency with the increase of AuNPs sizes. Finally the reusability of the AuNPs-SILLPs was evaluated by testing AuNPs-SILLP **160b**. The catalytic activity decreased for consecutive uses from 67% to 39%, 37% and 35% for the 2nd–4th cycles, because of the lixiviation of the active metal species from the stabilized NPs. In order to improve the applicability of these catalytic systems, a polymer cocktail containing the initial catalytic composite AuNPs-SILLPs **160b** along with the corresponding basic SILPs (R = Dec. X = OH–, macro, 3.45 mmol OH-/g), which acted simultaneously as a scavenger for the lixiviated species and as a base to modify the catalytic process, was evaluated. A positive effect was observed. The yield in the first cycle increased in comparison with the reaction performed in the absence of the basic SILLP (82% vs. 67%) and the catalytic activity decreased at a lower extent (73% for the 2nd, 58% for the 3rd and 45% for the 4th use). Finally, synthesis of some PdNPs-SILLPs was also performed. Under similar conditions, catalytic activity of AuNPs-SILLPs was to proved to be better than that of PdNPs-SILLPs.

**Scheme 62:**
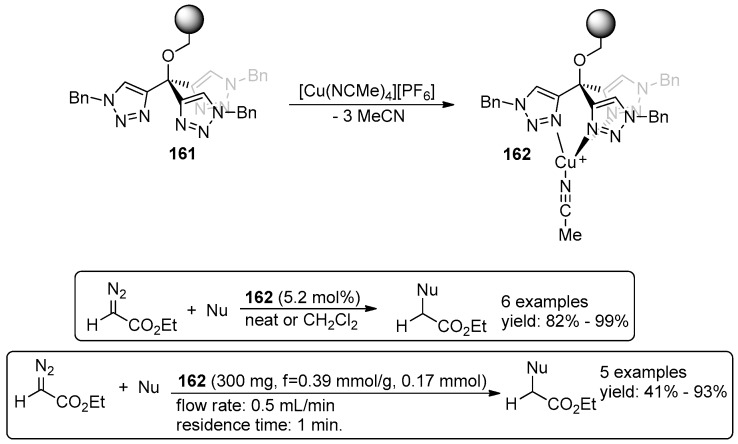
Preparation of polystyrene-linked tris(triazolyl)methanecopper (I) cationic catalyst **162** and its application in carbene transfer reaction in batch and flow conditions.

Recently, the polystyrene-linked tris(triazolyl)methanecopper (I) cationic catalyst **162** was reported for the general carbene transfer reaction in batch and flow reactions [103]. It was prepared by reaction between the polystyrene-supported tris(triazolyl) methyl complex **161** and [Cu(MeCN)_4_][PF_6_] in dichloromethane at room temperature (Scheme 62). It was applied, in 5.2 mol % loading, in the reaction of ethyl diazoacetate (EDA) with six substrates under batch conditions, obtaining high yields. The recyclability is evaluated in each reaction. The catalyst was separated by filtration and reused for five consecutive reaction cycles, keeping the reaction time constant. Essentially constant yields were observed; a slight decrease was noticed only with less reactive cyclohexane in the C–H bond insertion reaction. Furthermore, these six reactions were also carried out with a single catalyst sample in a sequential manner. Each reaction was repeated two times, and no decrease in activity was detected in twelve consecutive experiments. Then, the catalytic system was also investigated in continuous flow. Under optimized reaction conditions involving a flow rate as high as 500 µL·min^-1^ (equivalent to 1 min residence time), the preparation of small libraries of compounds was carried out by reaction of five different substrates with EDA. Four different types of carbene transfer (O–H insertion, N–H insertion, C–H insertion and cyclopropenation) in a sequential manner were carried out. Except for the cyclopropenation case, productivities in flow were significantly higher (up to four times) than those recorded for the same reactions in batch.

## 5. Metal-Catalysed Asymmetric Reactions

Supported metal-based catalysts can be also used in asymmetric transformations. In this section, two examples of metal-catalysed asymmetric C–C forming reactions, including the allylation of ketones and the Henry reaction, and three examples of asymmetric hydrogenation are described.

**Scheme 63:**
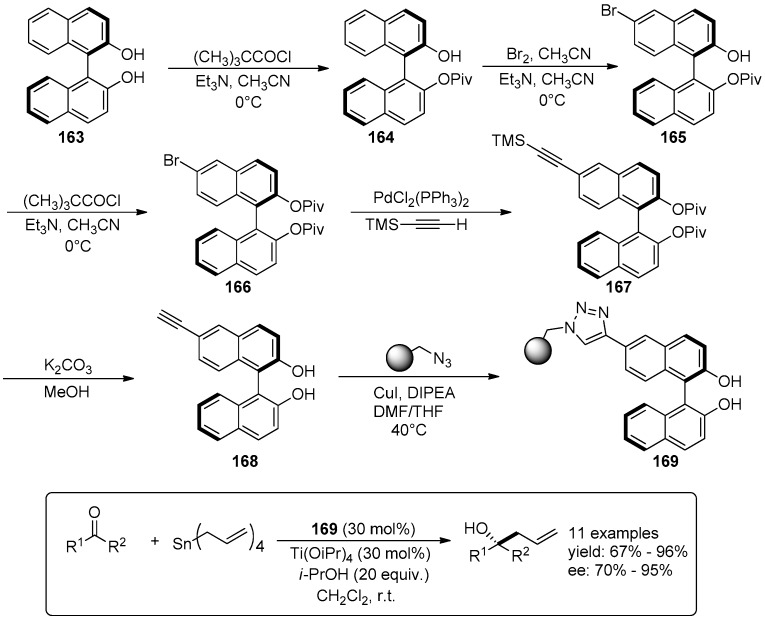
Synthesis the supported BINOL ligand **169** and its application in the titanium-catalysed asymmetric allylation of ketones.

Synthesis of the supported 1,1-binaphthol (BINOL) ligand **169** and its application in the titanium-catalysed asymmetric allylation of ketones were reported [104]. Supported BINOL ligand **169** was prepared by anchoring the enantiopure 6-ethynyl-BINOL **168** to an azidomethylpolystyrene resin through a copper-catalysed alkyne-azide cycloaddition reaction (Scheme 63). The enantiopure 6-ethynyl-BINOL **168**, having a suitable alkyne anchoring linker, was synthesized by the following route. The selective monoesterification of the hydroxyl groups of (*R*)-1,1–binaphthol (**163**) with bulky pivaloyl chloride was performed, to allow the successive chemoselective bromination at the 6-position and thus avoid the 6,6′-difunctionalization of the naphthyl groups. The obtained bromo derivative **165** was treated with pivaloyl chloride, and subsequently with trimethylsilylacetylene to introduce the trimethylsilylethynyl substituent, by a palladium-catalysed Sonogashira coupling reaction, and thus to furnish intermediate **167**. Finally, the hydrolysis and desilylation of latter intermediate by treatment with K_2_CO_3_ in methanol afforded the enantiopure 6-ethynyl-BINOL **168**. The supported BINOL ligand **169** was employed in the asymmetric allylation of a series of ketones with tetraallyltin under optimized conditions in presence of titanium(IV) isopropoxide and 2-propanol in CH_2_Cl_2_. The polystyrene (PS)-supported BINOL ligand **169** was converted into its diisopropoxytitanium derivative in situ and the asymmetric allylation mediated by the so-formed PS-supported (BINOLate)Ti species led to alcohols with high yield (67%–96%) and enantiomeric excesses (70%–95%). Recycling of the PS-supported (BINOLate)Ti species was investigated through the asymmetric allylation of 3-methylacetophenone. The catalyst was recovered by filtration and reused. A decrease in enantioselectivity was observed. It was established by ICP-MS analysis that up to 96% of its initial titanium content is leached from the resin in a single reaction cycle. Based on these results, the intercycle remetallation with titanium tetraisopropoxidewas carried out to obtain the effective reuse. By using this approach, both yield and enantioselectivity were essentially preserved after three consecutive reaction cycles. Supported BINOL ligand **169** was also employed in the tandem asymmetric allylation/epoxidation of (*E*)-2-benzylidenecyclohexanone and the tandem asymmetric allylation/Pauson–Khand reaction of 1-(2-(phenylethynyl)phenyl)ethanone, obtaining final products with high stereoselectivity in both cases.

**Scheme 64:**
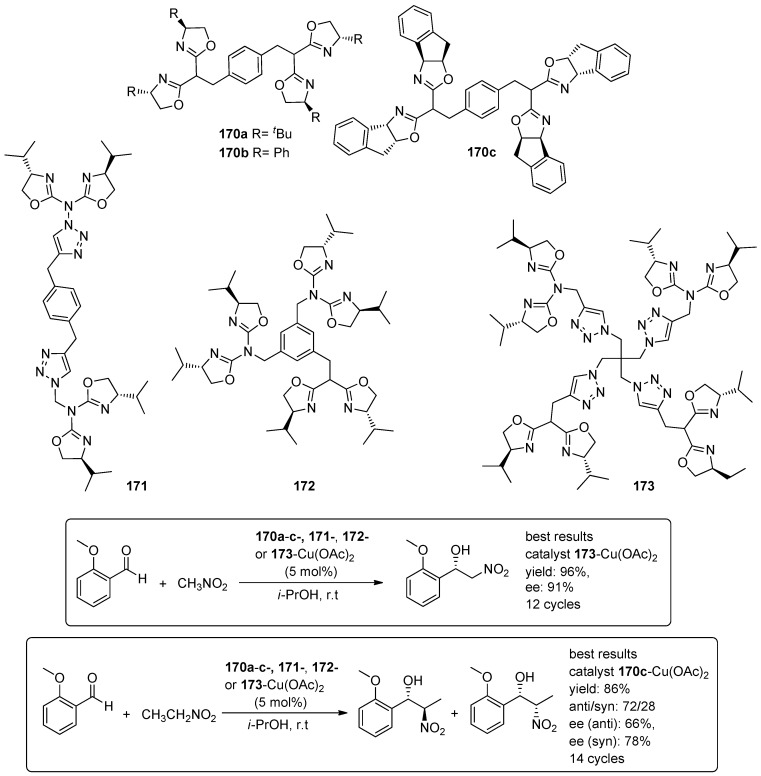
Henry reaction catalysed by coordination polymers **170**–**173**-Cu(OAc)_2_.

Several kinds of polytopic chiral ligands based on the bis(oxazoline) and azabis(oxazoline) motifs were used in the preparation of coordination polymers having copper as the ligand connecting metal [105]. Particularly, the ditopic ligands **170a**–**c** bearing *tert*-butyl, phenyl and indanyl substituents, respectively, the different ditopic ligand **171** bearing triazole units in the linker, the tritopic **172** and tetratopic **173** ligands were used (Scheme 64). The coordination polymers, formed by mixing the corresponding polytopic ligand (**170**–**173**) and Cu(OAc)_2_ in the appropriate Cu/Ligand molar ratio, were tested as recoverable catalytic systems for the Henry reaction of *o*-anisaldehyde with nitromethane or nitroethane. During the reactions, the polymers disassembled due to competitive coordination of either the reactants or the reaction solvent. The resulting soluble monomeric metal complexes acted as true homogeneous catalysts. At the end of each reaction, the release–capture strategy for the recovery and reuse of enantioselective catalysts, based on the precipitation of an insoluble coordination polymer, was shown to be effective in all cases. In the reaction between *o*-anisaldehyde and nitromethane, the best performances were obtained with ditopic ligand **171.** The use of the complex **171**-Cu(OAc)_2_ provided excellent results in both yield and enantioselectivity (96% and 91% respectively). It was reused up to 12 runs without loss of enantioselectivity and activity. In the reaction between *o*-anisaldehyde and nitroethane, the best performances were obtained with ligand **170c**. The **170c**-Cu(OAc)_2_ could be reused up to 14 runs with a slight decrease in yield, diastereo- or enantioselectivity.

**Scheme 65:**
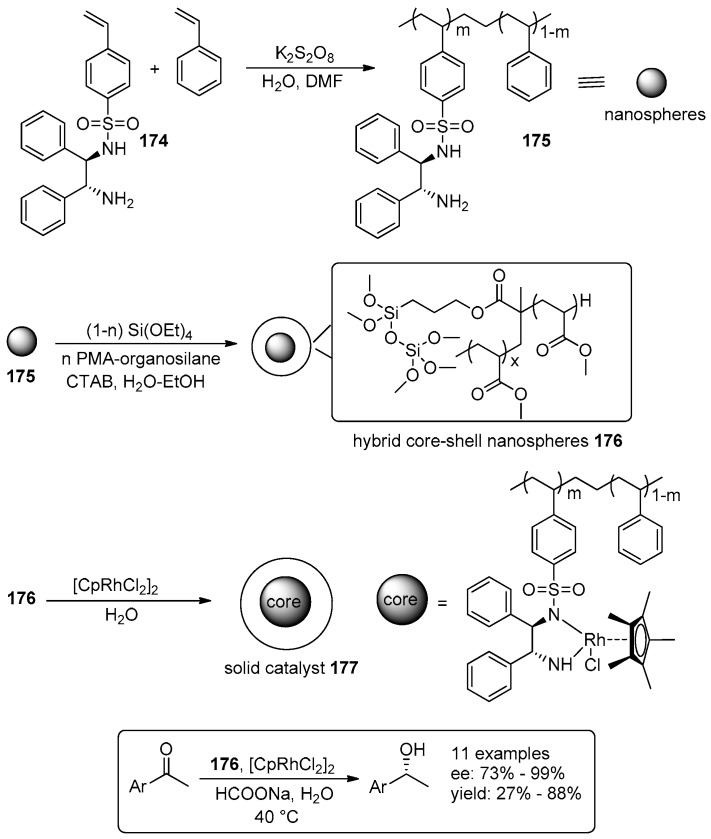
Preparation of the hybrid core–shell nanospheres **160** and its use in the Rh-catalysed ATH of aromatic ketones.

A new approach of fabricating core–shell structured solid catalysts for the asymmetric transfer hydrogenation (ATH) of aromatic ketones with [Cp*RhCl_2_]_2_ (Cp* = pentamethylcyclopentadiene) as metal precursor was investigated [106]. In detail, the hybrid core–shell nanospheres **176** with *N*-(*para*-toluenesulfonyl)-1,2-diphenylethylenediamine-polystyrene (TsDPEN–PS) in the core and poly(methyl acrylate)-functionalized silica in the shell were fabricated by two synthetic steps (Scheme 65). In the first step, TsDPEN–PS latex nanoparticles **175** were prepared through the emulsion polymerization of a mixture of chiral monomer **174** and styrene. In the second step (the coating process), the hydrolysis and condensation of the mixture of tetraethoxysilane and poly(methyl acrylate)-functionalized alkoxysilyl (PMA-organosilane), in presence of TsDPEN–PS latex nanoparticles **175** and cetyltrimethylammonium bromide (CTAB) as a structure-directing agent, were carried out.

The hybrid core–shell nanospheres **176** with different compositions and surface properties were tested in the Rh-catalysed ATH of acetophenone in aqueous HCOONa. The core–shell structured solid catalyst **177** was generated in situ by reaction between the hybrid core–shell nanospheres **176** and [Cp*RhCl_2_]_2_ (Cp* = pentamethylcyclopentadiene). The studies showed that the presence of CTAB and poly(methyl acrylate) polymer in the shell favoured high activity, which was mainly due to the increased surface hydrophobicity. Furthermore, the hybrid core–shell nanospheres **176**, obtained by using 10 mol % of the chiral monomer, in the process of the formation of the core, and 10 mol % of PMA–organosilane, in process of the formation of shell, were employed in the Rh-catalysed ATH of a wide range of simple aromatic ketones obtaining high enantioselectivities (ee: 73%–99%). The catalyst was separed through filtration and was recycled. Recycling study was carried out by ATH of acetophenone. After each run, the reaction time was prolonged to maintain the catalytic activity but the ee value of each run kept up well. The gradual decrease in the catalytic activity was due to the slight loss of Rh, which was confirmed by inductively coupled plasma analysis (5.4% in total Rh loading).

**Scheme 66:**
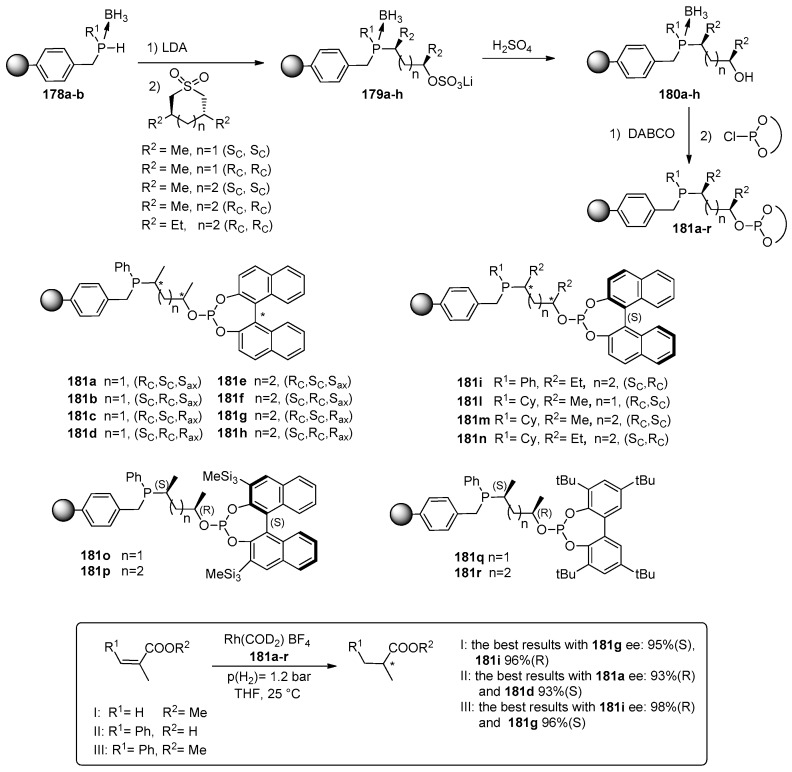
Synthesis of polymer supported phosphine–phosphite ligands **181a**–**r** and their application in rhodium catalysed asymmetric hydrogenation.

Recently, the modular solid-phase synthesis of a library of 16 supported phosphine–phosphite (P–OP) ligands, denoted as **181a**–**r**, and its application in rhodium catalysed asymmetric hydrogenation were reported (Scheme 66) [107]. Variations in structure of supported phosphine–phosphite (P–OP) ligands **181a**–**r** were generated by using phosphines bearing different substituents (R^1^), cyclic sulfates having a varying backbone length (n) and different substituents (R^2^) and by employing various chlorophosphites. The starting synthons of the solid-phase synthesis were supported phosphine–boranes **178a**–**b**, synthesized starting from Merrifield resin and primary lithium phosphides having different substituents (R^1^). Upon deprotonation of **178a**–**b** with lithium diisopropylamide (LDA), the lithiated phosphine–boranes reacted with a cyclic sulphate, affording compounds **179a**–**h**. Subsequently, the sulfate group was hydrolysed and the borane group removed by treatment with an excess of 1,4-diazabicyclo[2.2.2]octane (DABCO, 10 equiv.). Finally, the resulting hydroxyalkyl phosphines reacted with chlorophosphite reagents yielding supported phosphine–phosphite ligands **181a**–**r**. The library of 16 supported phosphine–phosphite ligands **181a**–**r** was employed in the asymmetric hydrogenation of three substrates. The complexation was performed prior to catalysis by suspending the resin-bound ligands in dichloromethane in the presence of rhodium precursor ([Rh(COD)_2_]BF_4_). In all cases, full conversion was achieved and enantioselectivities up to 98% were observed. The reusability of **181i** was assessed. It could be successfully reused for 11 reaction cycles with only a minor loss of activity and no decrease in selectivity.

**Scheme 67:**
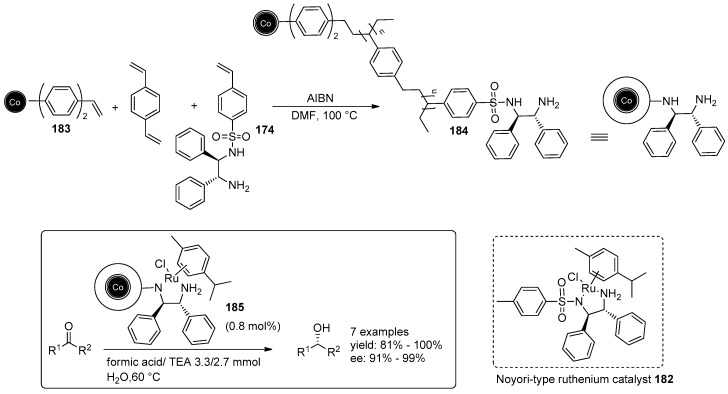
Synthesis of immobilized ligand **184** and its application in the Ru-catalysed ATH of aromatic ketones.

The synthesis and application of magnetic Noyori-type ruthenium catalysts for ATH reactions were reported [108]. Particularly, Noyori-type ruthenium catalyst **182** (Scheme 67) was immobilized on magnetic supports constituted by carbon coated cobalt nanoparticles (Co/C) and a polymer matrix. Among the supports prepared varying the polymer matrix and the linker, the immobilized ligand **184** gave the best results. This latter was performed by further functionalization of diphenyl vinyl functionalized nanoparticles **183**, via co-polymerization with vinyl substituted diamine ligand **174** and DVB in a ratio of 1:50:50 (Scheme 67). Thanks to the complexation in situ of ligand **184** with [RuCl_2_(*p*-cymene)]_2_, the active ruthenium catalyst, denoted as **185**, was obtained. In presence of **185**, a variety of aromatic ketones was reduced to their corresponding alcohols in an aqueous medium and with formic acid as hydrogen source. Good yields (81%–100%) and selectivities (91%–99% ee) were obtained. Furthermore, the heterogeneous catalyst **185** was recovered quantitatively by a magnetic decantation and reused 10 times showing high enantioselectivities and a gradual decrease in yield after the sixth run.

## 6. Conclusions

The immobilization of catalysts on polymer supports has proved to be a fundamental approach to realize recoverable and reusable catalytic systems. A great variety of strategies has been employed both in organocatalysis and in metal-based catalysis. Polystyrene-supported catalysts were prepared by grafting of an organocatalyst or a suitable ligand onto functionalized preformed polystyrene or, conversely, by copolymerization of styrene and/or divinylbenzene with a monomer containing the organocatalyst or ligand. Dendrimers immobilized on polymers have received great attention both as organocatalysts and as supports for metal catalysts. Furthermore, the application of poly(ionic liquid)s as heterogeneous catalysts allowed to overcome some drawbacks related to the use of relatively large amount of ionic liquid and the separation of products and catalyst. New organic-inorganic hybrid materials, including ionic liquid phase-based materials, were reported. Beside catalysts anchored as pendants groups on polymer support, new self-supported catalysts, in which the catalyst was incorporated in the main chain, were also described. However, the application of polymer supported catalysts, in some cases, has shown drawbacks such as decrease of catalytic activity and leaching of catalyst. This is a challenging field of research, which incites the design of new catalytic systems and new creative protocols for further improvement.
